# Synthesis and Applications of Selected Fluorine-Containing Fluorophores

**DOI:** 10.3390/molecules26041160

**Published:** 2021-02-22

**Authors:** Stefanie Casa, Maged Henary

**Affiliations:** 1 Department of Chemistry, Petit Science Center, Georgia State University, 100 Piedmont Avenue SE, Atlanta, GA 30303, USA; scasa1@gsu.edu; 2 Center for Diagnostics and Therapeutics, Petit Science Center, Georgia State University, 100 Piedmont Avenue SE, Atlanta, GA 30303, USA

**Keywords:** fluorine chemistry, optical fluorophores, PET imaging, PDT, contrast agents

## Abstract

The synthesis of fluorine-containing small molecules has had numerous benefits of improving the quality and efficiency of many applications of these compounds. For example, fluorine adds promising functionalities in various areas of imaging (MRI, PET, and NIR); gives cell-targeting properties; and has demonstrated improvements in cell permeability, solubility, and other pharmacologic properties. For these and other numerous reasons, fluorination of molecules has grown in popularity in various fields of chemistry. Many reports show the effects observed from increasing the number of fluorine atoms on a fluorophore scaffold. This report will cover the most significant applications and improvements that fluorine has contributed to in various dye scaffolds such as BODIPY, rhodamine, phthalocyanine, and cyanine in the recent decade.

## 1. Introduction

**Figure 1:**
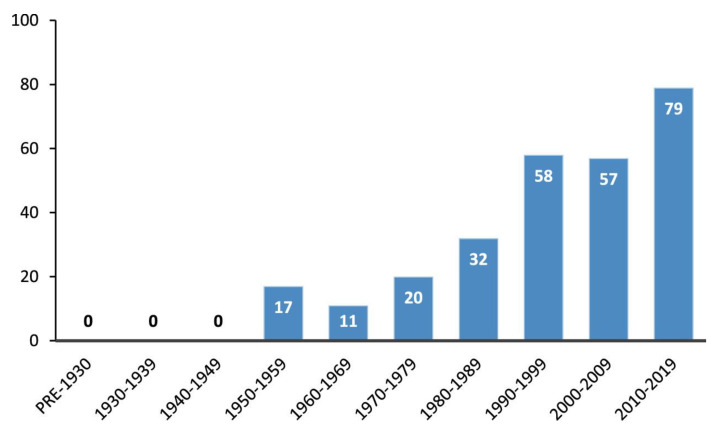
Fluorine-containing drugs being introduced into the market in previous years until 2019. Figure used with permission from [3]. Copyright 2020 American Chemical Society.

Fluorine-containing compounds have grown in popularity for medicinal chemistry, with some of the most recently popular drugs containing fluorine atoms: Fluoxetine, Atorvastatin, and Lansoprazole [1]. It is known that about 20% of commercialized medicines in the pharmaceutical industry have a fluorine atom [2]. It is also expected that fluorination of molecules will continue to play a significant role in the future of medicinal chemistry based on the current rate of fluorinated medications being introduced into the market (Figure 1) [3,4].

A significant reason for incorporating fluorine into a chemical structure is related to the size and properties of the fluorine atom. Of the halogen atoms on the periodic table, fluorine is the smallest in the group, and it is the most electronegative of the halogens. In these compounds, fluorine demonstrates electron-withdrawing effects, molecule stability, and enhanced chemical interactions [5,6]. The most relevant biological effects of fluorination being studied are drug metabolism, excretion, and ligand-binding interactions [7].

Fluorine has a small atomic radius (50 pm), comparable to a hydrogen atom (25 pm) and smaller than the other halogens: chlorine (100 pm), bromine (115 pm), and iodine (140 pm) [5,8]. Compounds with fluorine substituents also contain lipophilic properties with some literature reporting an increase in cell penetration observed in compounds after fluorine substitution, thus introducing a beneficial functionality for bioimaging and clinical studies [9,10]. Fluorine is also considered a beneficial atom to consider in drug design due to its ability to delay drug metabolism; most notably in lipophilic aromatic structures, which reduce toxicity by delaying oxidation of the drug allowing for improved excretion of drugs [11].

In fluorophore chemistry, halogens are typically sought after for their chemical properties (i.e., reactivity, electronegativity, lipophilicity, bond stability) on dyes; however, a significant amount of literature focuses on the halogen effects of chlorine and bromine on certain dyes, making them useful for therapeutic applications [9]. The halogen effect of fluorine, as well as other halogens, is significant in the design of dyes because it is observed that halogen substituents have different imaging and targeting effects biologically than other substituents; a prime example being the use of fluorine-exchange in dyes for positron emission tomography (PET) imaging [12,13]. The electron-withdrawing nature of the halogen atoms also exhibits repulsive and attractive effects in electrostatic interactions and alteration of reaction rates and molecular stability due to the strength of the carbon–halogen bond [14].

In medicinal chemistry, the substitution of specific functional groups with C-F has been considered an area of focus for biologically active molecules [13]. As will be seen in some examples, the presence of the fluorine atom enhances biological activities and functions as a targeting moiety for enzyme recognition [15,16]. These functions, as well as imaging functionalities, will aid in improving the activity of these classes of fluorophores. The fluorophores described below have different advantages and disadvantages; however, fluorination can show differing effects in each class to combat an obstacle specific to the class of fluorophore.

In dye synthesis, compounds in the field are used for different methods of analysis, detection, and functions. Fluorophores in the visible range optical window show peak absorption and emission values within 400 nm and 600 nm. Compounds such as derivatives of boron-dipyrromethene (BODIPY), rhodamine, and fluorescein typically demonstrate signal wavelengths around 500 nm and 600 nm; however, it is more favorable to see these signals closer to 700 nm to improve biological applications [17,18,19,20,21]. For this reason, recent studies described further in the text demonstrate the applications of adding fluorine-based substituents and phenyl rings to the chemical designs, thus resulting in greater absorption and fluorescence wavelengths.

**Figure 2:**
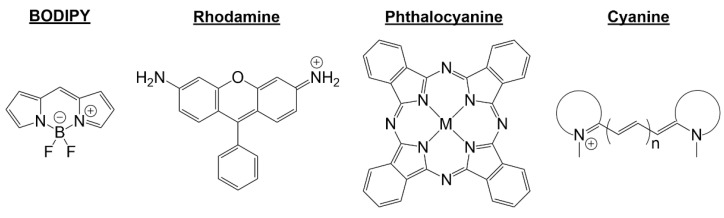
Fluorophore scaffolds; BODIPY, rhodamine, phthalocyanine, cyanine.

The structure of BODIPY is mainly unique due to the chromophore having a fluorinated boron center (Figure 2). Literature focuses on the synthesis of symmetric and asymmetric versions of BODIPY dyes using different alkyl groups, halogen atoms, and extended conjugation [17,18]. It is also essential to recognize the literature detailing substitutions of the fluorine atoms on the boron center [22,23]. These types of substitutions create or add different functionalities to the BODIPY scaffold. Some of these functionalities include enhanced fluorescence imaging, improved biomolecular targeting, and chemical/metal sensing as described in data.

Current synthesis trends mostly focus on pushing absorption and emission maximum signals further towards the 700–800 nm region to make them more optically relevant as fluorophores. Although this is mainly achieved with the addition of aromatic moieties to increase conjugation from the BODIPY core, the addition of fluorine atoms can assist in combating solubility issues that are typical of these planar and hydrophobic compounds [23].

The structure of rhodamine is unique due to the three fused-ring backbone. This branch of dye chemistry belongs to a group referred to as xanthene dyes [24]. Current synthesis focuses on the substitution of the oxygen in the center fused ring with silicon to produce more redshifted dyes of this scaffold and use fluorine to increase wavelength [25]. Literature also reports the use of different fluorine-containing alkylation reactions on the nitrogen groups and their effects on reaction rate and targeting enhancements.

The near infrared (NIR) region on the electromagnetic spectrum is within 700 nm and 1200 nm. The first near-infrared region is detailed in the 650 to 900 nm NIR I window, detailing some well-known classes of fluorophores that primarily absorb and fluoresce in this region. The compounds discussed in this region are phthalocyanine (around 670 nm) and cyanine (650–900 nm), as seen in Figure 2 [26].

Some of the most significant applications of phthalocyanines are in NIR imaging and photodynamic therapy [27]. Phthalocyanines can act as contrast agents due to their wavelength absorption being within the NIR I optical window, allowing them to be advantageous agents for fluorescence imaging. In photodynamic therapy (PDT) studies, phthalocyanines have been useful for their ability to undergo energy transitions that generate singlet oxygen that further becomes reactive with other biomolecules [28]. These intercellular side reactions cause disruptions in cell functions and can trigger cell death.

The most known shortcomings of phthalocyanines are their levels of solubility, making them less advantageous for in vivo studies and their susceptibility for aggregation, resulting in aggregation-induced quenching, presenting limitations of optical properties. These shortcomings are associated with the planar structure of the phthalocyanine core; the planar molecules experience pi-stacking at certain concentrations leading to aggregation [29]. However, researchers are working with different combinations of substituents appended to the phthalocyanine core to prevent this aggregation effect while also further improving imaging properties.

Cyanine dyes, as seen in Figure 2, have been a useful class of fluorophores for many years [30]. Researchers have searched for ways of synthesizing different versions of these dyes at different wavelengths; some might notice the distinct differences in optical properties between the three most reported categories: trimethine, pentamethine, and heptamethine [31]. Although a significant amount of synthesis has been reported about the effects specific moieties have on these compounds, much of the research left to study is improving upon the specificity of these compounds as well as improving optical properties.

In literature, cyanine dyes are designed with fluorine atoms in different positions to achieve desired optical properties and targeting functionalities. Some fluorine is added in the center bridge to improve cell targeting [32,33]. Some compounds show organ targeting or enzyme specificity after the introduction of fluorine atoms to the chemical structure. Like in other compounds, it is observed that the introduction of fluorine opens opportunities for other imaging modalities such as PET imaging [34]. Many new cyanine dyes are also being conjugated to targeting ligands for improved specificity.

This review will focus on the synthesis and effects of fluorine atoms on BODIPY, rhodamine, phthalocyanine, and cyanine dyes. The literature focuses mostly on introducing single fluorine atoms, trifluoromethyl groups, and fluorous carbon chains affecting the optical and biological properties of the scaffolds described in Figure 2. Although there are many examples of fluorine atoms improving the qualities of these fluorophores, it is vital to consider the trends in what has been achieved in recent years. Some of these modifications have achieved significant results that can inspire future research to apply similar changes to other dye scaffolds.

## 2. Fluorinated BODIPY Dyes

**Scheme 1:**
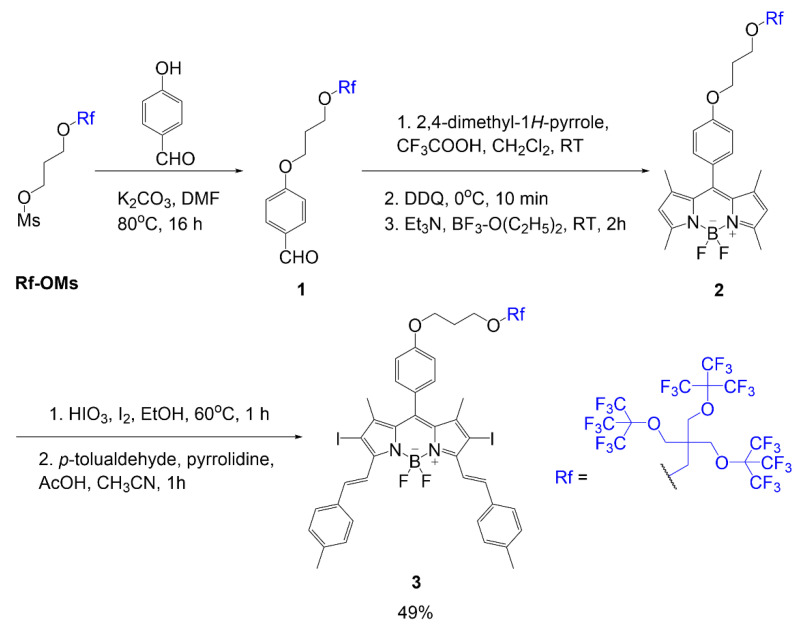
BODIPY with multi-branched fluorinated chains on meso phenyl [21].

Martinez Espinoza et al. studied the synthesis and effects of branched fluorinated chains off a BODIPY scaffold [21]. The synthetic design focused on the fluorinated chain placement in two different positions. One position was associated with an alkoxy group of the meso position of the dye, as seen in compound **3** (Scheme 1). The scheme begins with a substitution reaction between 4-hydroxybenzaldehyde reacting with fluorinated mesylate (Rf-OMs) under basic conditions to generate aldehyde **1**. Using this intermediate, a fluorinated BODIPY **2**, is formed in three steps typical of BODIPY scaffold formation. The following step is iodination to add iodine atoms to the BODIPY core; this modification is described to promote singlet oxygen generation in the final compound. The step after iodination adds two equivalents of *p*-tolualdehyde to the scaffold through Knoevenagel condensation to extend the conjugation of the final compound **3** with a yield of 49%.

**Figure 3:**
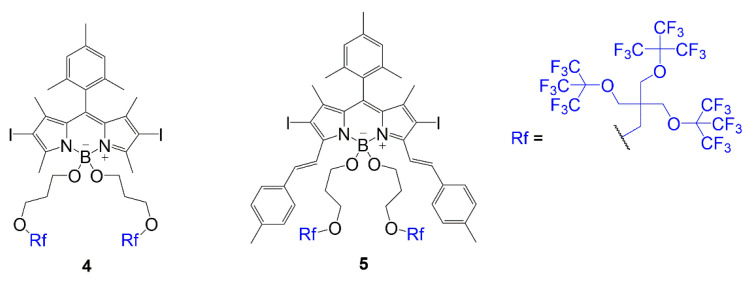
BODIPY with multi-branched fluorinated chains attached to boron center [21].

The other placement was a substitution of fluorine atoms at the boron center with alkoxy groups containing the fluorinated chain as seen in compounds **4** and **5** (Figure 3) [21]. In these dyes, an iodination step occurs to add iodine atoms to the BODIPY core to promote singlet oxygen generation. Both compounds contain a substitution to gain the fluorinated branches; however, compound **5** undergoes Knoevenagel condensation to extend conjugation before substituting the fluorine atoms. The structural differences in compounds **4** and **5** compared to compounds **2** and **3** are important to consider when determining the bathochromic shift caused by the substitution of the fluorinated chains because it is important to understand the impact of the fluorinated chains with and without the extended conjugation.

Another example of fluorine substitution on the boron center is viewed in the synthesis conducted by Duran-Sampedro et al. (Equation (1)). Trifluoroacetoxy groups replace both fluorine atoms [22]. The synthesis was accomplished to demonstrate the enhanced fluorescent effect of trifluoroacetoxy compared to a BODIPY with a standard core. This substitution method requires reagent TMSOCOCF_3_, which is synthesized using trifluoroacetic acid and TMSCl in 1,2-dichloroethane, to react with individual compounds **6**–**8**. The reaction affords solid compounds **9**–**11** (yields 22–37%) with fluorine demonstrating halogenic effects in the boron center of the BODIPY.


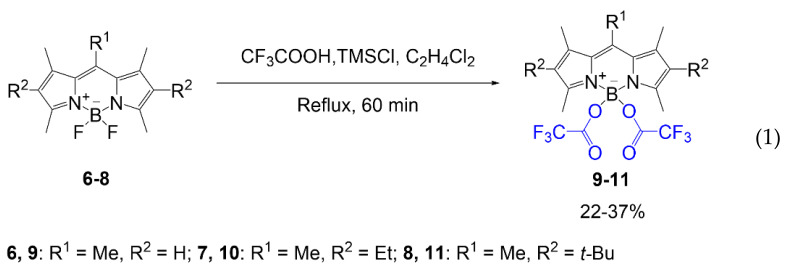


**Scheme 2:**
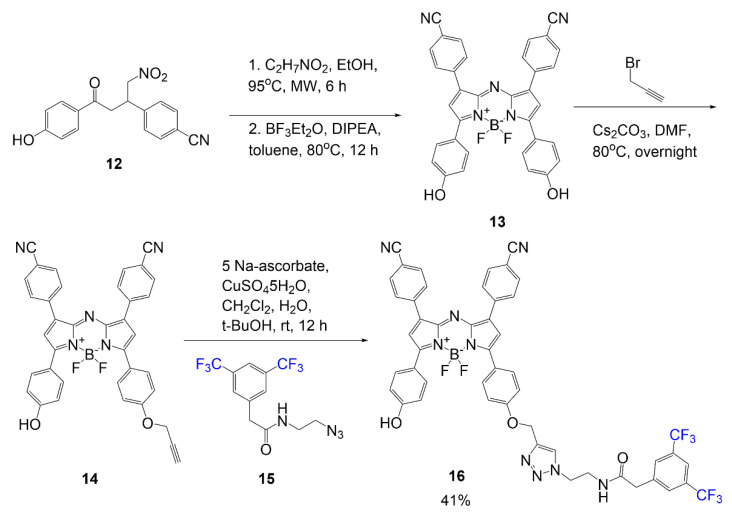
Synthesis of fluorinated trimodal BODIPY **16** [35].

In the next example, the aza-BODIPY **16** was synthesized as a trimodal contrast agent designed for fluorescence imaging, photoacoustic imaging, and ^19^F MRI [35]. Of the listed modalities, the BODIPY core demonstrates extended conjugation for enhancing fluorescence and photoacoustic imaging, and the trifluoromethyl groups on the aromatic ring are designed for ^19^F MRI functionality. In Scheme 2, the synthesis of contrast agent **16** begins with the ring closure of the BODIPY using compound **12** with ammonium acetate in ethanol under microwave conditions; the second step completes the closure using a boron-source to yield BODIPY **13**. A nucleophilic substitution reaction occurs between compound **13** and 3-bromo-propyne to form compound **14**. Through click chemistry, alkyne **14** forms a five-membered triazole ring with azide **15** to yield final compound **16** in a good yield of 41%.

**Scheme 3:**
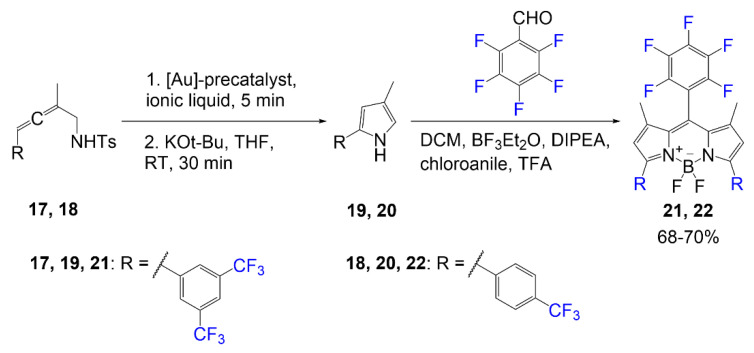
Fluorinated BODIPY synthesized via gold catalyst [36].

Scheme 3 illustrates the synthesis of fluorinated BODIPY synthesized using a gold catalyst for the cycloisomerization of the fluorine-containing pyrrole precursor [36]. In previous literature, research groups have been studying the use of aromatic groups to extend conjugation of BODIPY dyes [17]. In this study, they synthesize pyrrole rings with aromatic rings containing trifluoromethyl group(s). The first reaction shown in Scheme 3 is the two steps done on compound **17** or **18** to form the pyrrole **19** or **20**. The first step demonstrates the use of the gold catalyst in ionic liquid, and the second step shows the basic conditions to finalize the ring closure. After generating the relevant pyrrole, the BODIPY scaffold is synthesized using the pentafluorobenzaldehyde and pyrrole **19** or **20** with BODIPY reagents: chloroanile, BF_3_·Et_2_O, and DIPEA; this reaction generates product **21** or **22** in good yield 68–70%.

**Scheme 4:**
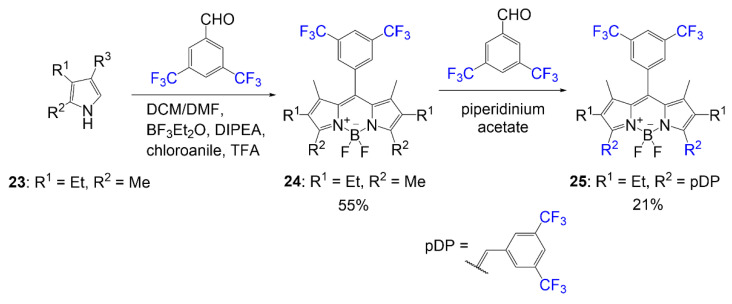
Synthesis of BODIPY containing fluorinated aryl groups [37].

In an effort to design a BODIPY dye with higher photostability, Hecht et al. synthesized a series of fluorinated BODIPY dyes and fluorinated BODIPY dyes with extended conjugation of the alpha-3 and alpha-5 positions [37]. As shown in Scheme 4, compound **25** was synthesized using the general BODIPY procedure to obtain compound **24**, followed by a Knoevenagel condensation using a fluorine-containing benzaldehyde. The Knoevenagel condensation is achieved utilizing a benzaldehyde derivative and a piperidinium acetate catalyst. This conjugation extension is used to shift BODIPY absorption values to higher wavelengths. The yield reported for the synthesis of dye **25** is 21%.

**Figure 4:**
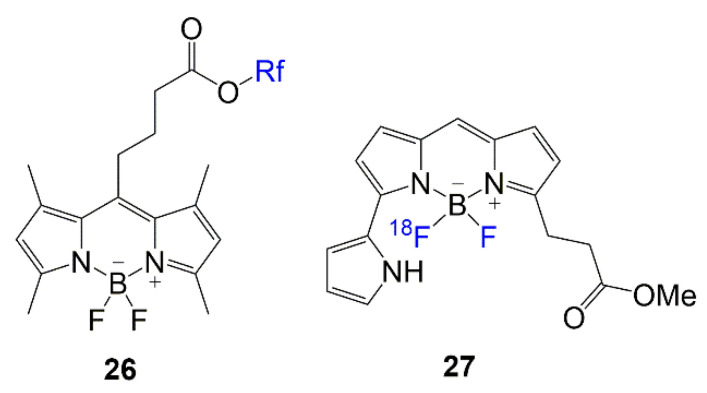
Fluorinated BODIPYs used in ^18^FMRI and PET imaging modalities [38,39].

Lastly, in Figure 4, the chemical structures of BODIPY compounds **26** and **27** are displayed. These compounds are relevant in recent literature for their biological applications. The design for dye **26** is similar to the rationale of compounds **2**–**5**, with multi-branched fluorinated chains being the focus of the molecule [38]. The synthesis of dye **27** mostly addresses the fluorine exchange to generate the ^18^F-labeled BODIPY to introduce the PET modality to the scaffold for brain imaging [39].

### 2.1. Optical Properties of Fluorinated BODIPY

BODIPY compounds typically demonstrate absorption spectra with a peak around 510 nm, typically more intense and relevant as a visible/NIR contrast agent. Newer dyes in literature have aryl groups in different positions of the BODIPY dye to afford shifts in wavelength that will improve in vivo applications. The most common is having a phenyl group at the meso position. Studies were conducted to view the effect fluorine atoms have on BODIPY when fluorine is observed in the meso phenyl at different placements [23]. These studies have indicated increasing absorbance values associated with increasing fluorine atoms in the molecule. They have also noted shifts in absorbance wavelength due to the position of the fluorine atoms in proximity to the BODIPY core. The absorption shift and increasing quantum yields are typically observed in compounds with halogens; however, the halogen’s position will affect the degree to which the increase is experienced. Table 1 indicates the optical properties of BODIPY fluorophores shown in Scheme 1, Scheme 2, Scheme 3 and Scheme 4, Figure 3 and Figure 4, and Equation (1) with fluorine-containing functional groups/atoms at differing positions.

Substitutions of fluorine on the boron of the BODIPY for trifluoroacetoxy causes a shift in peak absorbance wavelength [22]. Compared to the compounds created from the substitution of fluorine for acetoxy groups, compounds **9**–**11** demonstrate slightly blueshifted absorbance values, 502–532 nm; however, it demonstrates redshifted optical values compared to precursors **6**–**8** detailed in the literature. Quantum yield of compounds **9**–**11** does increase from the presence of fluorine in the trifluoroacetoxy groups compared to both the commercially available BODIPY **6**–**8** and the nonfluorinated versions of these fluorophores.

In the example of the trimodal BODIPY, compound **16** was synthesized [35]. As expected, the addition of the component containing the trifluoromethyl groups did not afford a bathochromic shift as seen in other compounds; however, this was not expected due to the proximity of the fluorine to the BODIPY core. A significant increase in molar absorptivity, 59,815 M*^−^*^1^ cm*^−^*^1^, and quantum yield 42% were notably observed, like other fluorine-containing compounds.

Compounds **21** and **22** are similar in structure to compound **25**. They contain a fluorinated phenyl group in the meso position, either pentafluorophenyl or trifluoromethyl groups on phenyl. When comparing compounds **21** and **22** to compound **25**, the fluorinated phenyl affords a 10–16 nm shift. Most of the newer compounds in literature utilize a combination of increasing the conjugation of the BODIPY and increasing the number of fluorine atoms in the molecule to increase molecule absorbance around 30–200 nm. This shift in the wavelength offers the potential to push the synthesis of BODIPY compounds into the NIR optical window to improve the properties of these molecules applied as contrast agents without making them too large or complex.

### 2.2. Applications of Fluorinated BODIPY

Literature explores the lipophilic effect that is caused by fluorine atom in BODIPY dyes [23]. The fluorinated version of the BODIPY compounds is comparable to the version of the structure with hydrogen in the place of fluorine due to the compound’s size while offering electron-withdrawing halogenic effects to small molecules. This difference allows research to be done to compare hydrogen and fluorine atoms to explore the impact of fluorine atoms on BODIPY based on the quantity and position of atoms. Some of the most important studies to occur are the observations of fluorine substitutions on the center boron and observing fluorine effects on aromatic carbons.

**Figure 5:**
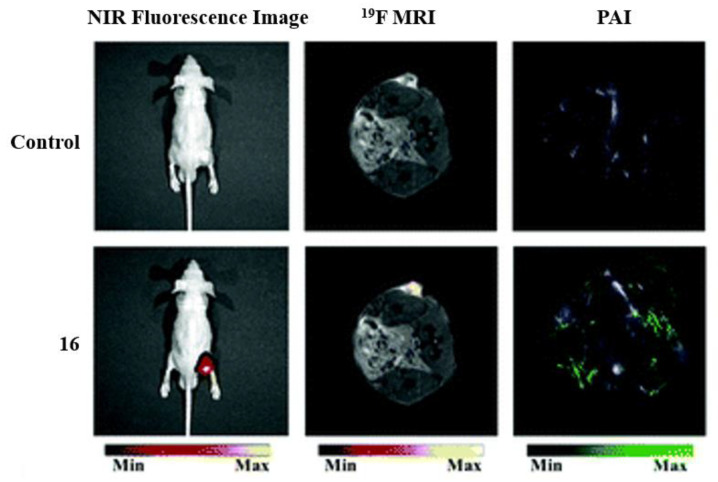
Applications of fluorinated trimodal compound **16**, for in vivo imaging. Imaging of an A549 tumor in the mouse model. Before injection (control, **top**) and after injection (**bottom**) demonstrating the three imaging modalities: NIR fluorescence (**left**), ^19^FMRI (**middle**), and photoacoustic imaging (PAI) (**right**). Figure used with permission from [35]. Copyright 2019 Royal Society of Chemistry.

Many well-known BODIPY dyes have been utilized for medical imaging. BODIPY dyes can be used as fluorescent switches and sensitizers for metal/pH sensing and being designed for specific cell or biomolecule targeting [18]. Although BODIPY compounds are typically hydrophobic compounds, which is a disadvantage for them to be utilized for biological applications, the addition and placement of the fluorine atoms can help overcome hydrophobicity [17]. In the example of Figure 5, dye **16** is used as a trimodal contrast agent because, in acidic conditions, the agent will exhibit fluorescent properties [35]. Fluorescence imaging alongside photoacoustic imaging (PAI) and ^19^FMRI allow for the activity of the compounds to be enhanced for tumor imaging and analysis. The fluorescence imaging shows the dye localizing to the tumor, while ^19^FMRI and PAI imaging show sharp signals for the tumor cells.

**Figure 6:**
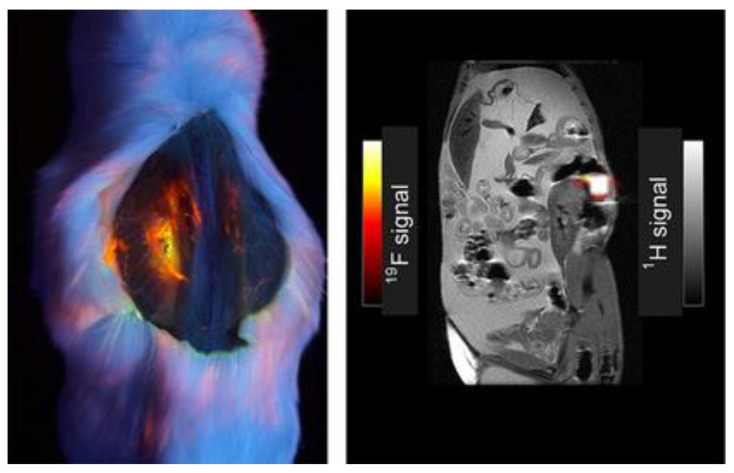
Dual fluorescence and ^19^F MRI, dye **26** (100 µL of 100 mM in DCM). Image under black-light excited at 365 nm of mouse post-mortem (**left**); grayscale image (**right**) demonstrating overlay of ^1^H and ^19^FMRI. Figure used with permission from [38]. Copyright 2016 John Wiley & Sons.

The introduction of fluorine atoms in these molecules shows improvements in the specificity of signal in certain forms of imaging. Figure 6 shows imaging comparing ^19^F signal to ^1^H signal in a mouse model injected with 100 mM of compound **26**, and the intensity of the fluorine signal in one location is appreciable [38]. The signal shows relatively high intensity for the fluorophore pictured (left); this compound has a similar structure to BODIPY compounds **2**–**5** featuring the multi-branch fluorination (Scheme 1). This observation is important to consider since the ^19^F signal is very bright and localized to one area of the abdominal cavity, making it worth further investigating multi-branched fluorinated chains on BODIPYs.

**Figure 7:**
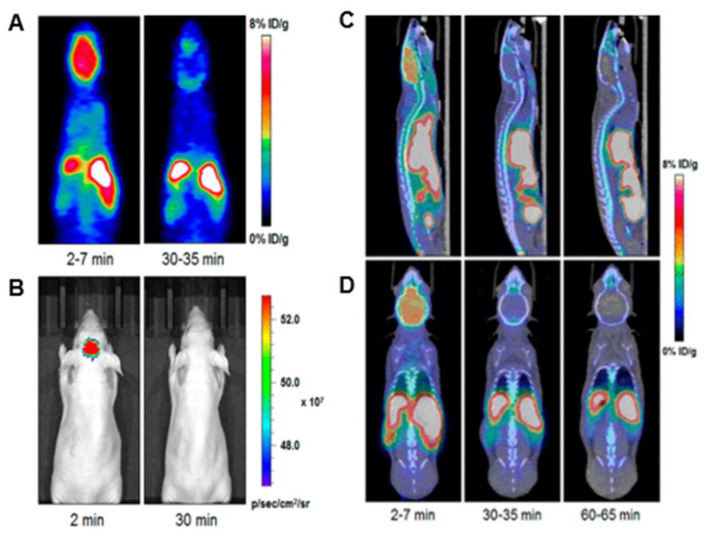
Radioactive fluorine exchange in BODIPY used for PET, compound **27**. (**A**) PET imaging and (**B**) optical imaging was done at two intervals: 2 min and 30 min (0.1 GBq/µmol). (**C**,**D**) indicate MicroPET/CT images at the same intervals with an additional image at 60 min; (**C**) sagittal view and (**D**) coronal view (0.8–1 GBq/μmol). Figure used with permission from [39]. Copyright 2019 American Chemical Society.

One of the most interesting uses of these fluorine-containing BODIPYs is its applications in Positron Emission Tomography (PET) imaging [39,40]. Figure 7 demonstrates imaging after injection of compound **27** into a mouse model. It is observed that strong signals are observed in the brain upon 2 min of injection and diminishes in the images after 30 min. Although this is a short amount of time for a dye, this is an impressive achievement as many groups are pursuing brain-targeting probes. Although other compounds possess the potential for this type of application, these BODIPYs with the active fluorine substituted onto the boron have shown some of the most reports for PET imaging data in the literature, making fluorine-exchange on the boron well-known in BODIPY synthesis [39]. This functionality continues to be a growing interest in BODIPY research with continued improvements in the solubility of these compounds and the penetration of the blood–brain barrier.

Some literature recognizes the potential for fluorinated BODIPY compounds to be used in cell imaging due to their lipophilic character having the potential for cell membrane staining [36]. The literature addresses the tremendous potential fluorine atoms cause for fluorescence imaging through improvements in molar absorptivity, increased fluorescence efficiency, and increased lasing efficiency [22]. However, most of the literature emphasizes the improvements in optical properties above all else on the effects fluorine has on BODIPY.

## 3. Fluorinated Rhodamine Based Dyes

**Scheme 5:**
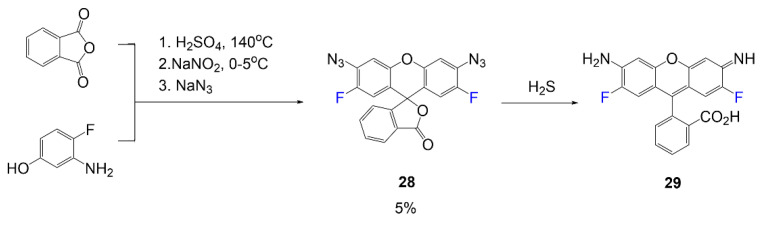
Hydrogen sulfide sensing rhodamine dye [41].

In a work by Wei et al. in Scheme 5, rhodamine dye sensitive for detecting hydrogen sulfide in biological environments was synthesized [41]. The initial fluorescent rhodamine, compound **29**, is synthesized in a one-pot reaction mixture utilizing 3-amino-4-fluorophenol and o-phthalic anhydride in acid. In the following two steps, the compound is transformed into the nonfluorescent lactone form of the rhodamine **28** through a Sandmeyer reaction using sodium nitrite and sodium azide in consecutive steps. It has been reported that the electron-withdrawing fluorine atom demonstrated a faster reaction rate. When in conditions of hydrogen sulfide, rhodamine **28** will convert to the fluorescent form, product **29**. Low yield is reported for compound **28** (5%), while yield is not reported for the fluorescent form, dye **29**.

Rhodamine dyes **33**–**35** are synthesized as targeting small ligands for intracellular protein labeling. As seen in Equation (2), compounds **30**–**32** are treated with fluorinated azetidine rings. They utilize fluorine substituents that manipulate equilibrium between the lactone and fluorescent forms of the dye and improve quantum yields [42]. Further studies demonstrate this same fluorine effect using a different center atom in the rhodamine core. The change in the rhodamine core creates various possibilities for redshifted rhodamine dyes.


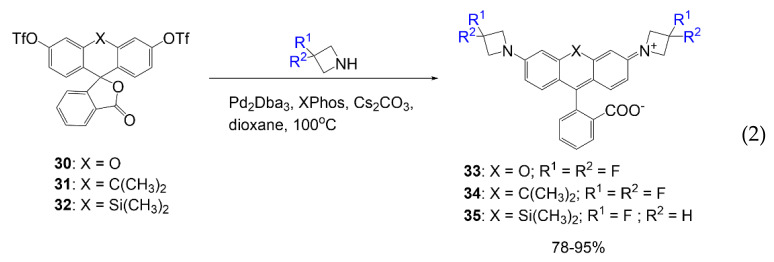


Dye 37 is designed by Wan et al. to be utilized as a Schiff base chemosensor for aluminum (III) ions [43]. As seen in Equation (3), compound **36** reacts with 4-fluorobenzaldehyde in methanol at high temperature to synthesize product **37** in a good yield of 85%. In this compound, the fluorine atom is designed into the rhodamine to improve the selectivity of the metal ion.


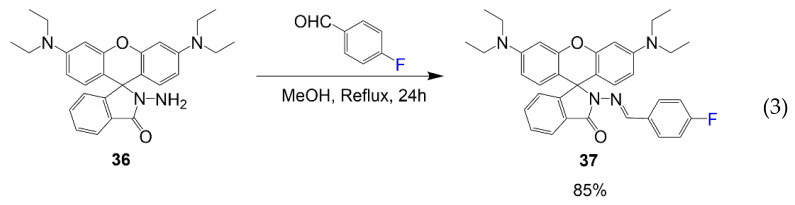


**Figure 8:**
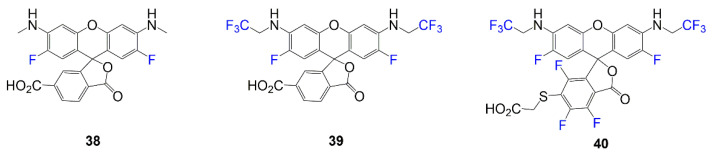
Precursors to targeting probes [44].

Rhodamine dyes **38**–**40** are synthesized through general synthesis requiring the corresponding version of phthalic anhydride and the appropriate fluorinated phenol under basic conditions. Figure 8 shows the simple fluorinated rhodamine precursors **38**–**40**. Optical properties for these compounds are reported, and the rhodamines undergo further reactions to form an amide bond at the carboxylic acid functional group, thus attaching a targeting molecule to synthesize the functionalized probe [44]. At the carboxylic acid end, lysosome probes utilize Pepstatin A for targeting, and mitochondria probes use (4-Carboxybutyl)triphenylphosphonium.

**Scheme 6:**
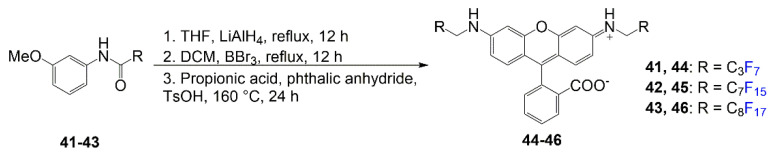
Synthesis of rhodamine F derivative [45].

Compounds **44**–**46** below demonstrate the increasing lipophilic characteristics of fluorinated chains on rhodamine to show a relationship between fluorophilicity and lipophilicity [45]. This class of rhodamines are referred to as rhodamine F as they exemplify fluorous chains bonded to the nitrogen atoms. In Scheme 6, compounds **41**–**43** undergo a reduction using a reducing agent, lithium aluminum hydride, to convert the carbonyl to a sp^3^ carbon. The second step is demethylation of the methyl on the methoxy group using boron tribromide to achieve. After the two steps, the relevant phenol reacts with phthalic anhydride under acidic conditions to afford the final compounds **44**–**46**. Yields for dyes **44**–**46** are not reported.

**Scheme 7:**
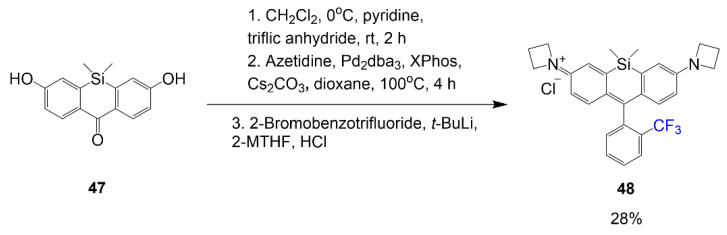
Synthesis of trifluoromethyl-containing si-rhodamine [46].

A fluorinated Si-rhodamine is observed to have redshifted absorption spectra over the nonfluorinated compounds [46]. In Scheme 7, the Si-rhodamine is synthesized with a phenyl ring containing a trifluoromethyl substituent compared to a similar dye with a methyl substituent. The first step includes a reaction mixture of compound **47** and triflic anhydride in dichloromethane under basic conditions to achieve the triflation. The second step details the conditions to substitute the azetidine rings onto the xanthone in the place of the oxygen atoms. The final step introduces the pendant phenyl ring with the trifluoromethyl group in the carbonyl site through nucleophilic addition to afford the final product **48** with a yield of 28%.

**Scheme 8:**
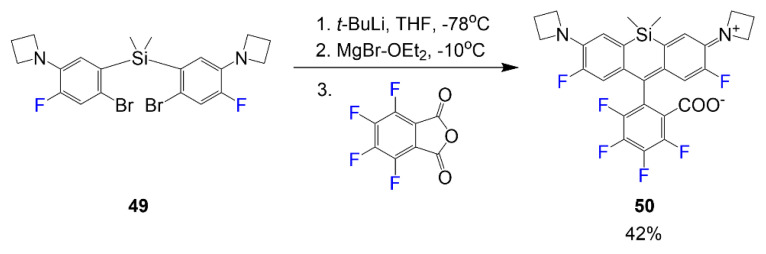
Synthesis of fluorine-containing si-rhodamine [47].

The reaction shown in Scheme 8 undergoes a series of reactions beginning with a Li/Br exchange of compound **49** in the first step using an excess amount of tert-butyl lithium in THF [47]. The following step requires a transmetalation reaction to occur using MgBr_2_·OEt_2_ before finally adding the phthalic anhydride electrophile in the third step to synthesize dye **50** in a yield of 42%.

Although there are some examples of fluorinated rhodols and other dye scaffolds that fall within the xanthene dye family, fluorinated rhodamines have been showing the most abundance of reported synthesis in the last few years [48]. This observation is likely due to amino groups on the scaffold demonstrating stronger optical properties than other scaffolds in this family. This is a very important observation to consider in dye chemistry as promising optical properties have the potential to make the compounds more relevant for biological applications.

### 3.1. Optical Properties of Fluorine-Containing Rhodamine Dyes

Rhodamine dyes typically fluoresce at wavelengths within the visible range. The majority of the new synthesis of rhodamines considers methods to synthesize dye at higher wavelengths to achieve fluorescence towards the NIR region. Halogen substituents and the introduction of a silicon atom are typically used to accomplish this bathochromic shift in different ways. Table 2 demonstrates the selected rhodamine compounds that have been chosen to illustrate the varying effects the fluorine atoms have in different positions of the rhodamine core and the changes in optical properties observed.

Table 2 highlights the bathochromic shifts presented by the different fluorinated rhodamine dyes. Some of the most popular modifications made to the rhodamine structure are the addition of alkyl groups to the nitrogen of rhodamine, most commonly adding an azetidine ring or fluorine-containing alkyl groups as seen in compounds **38**–**40** [42,43,46]. Fluorine atoms are also typically incorporated into the rhodamine structure on the carbon adjacent to the amino group like in compound **29**, or on the phenyl ring added by the phthalic anhydride precursor as shown in compound **48** or **50**. Although most of these additions are expected to red-shift the absorption/fluorescence signals, some combinations of these additions do not achieve that.

Initially, the absorption of compound **29** is used to compare the effect noticed by the addition of alkyl groups to the free amino groups as seen mainly for compounds **33**–**35**. Compound **29** is considerably lower in absorbance than most of the other compounds reported in Table 2. From the 488 nm absorption, a significant bathochromic shift is observed based on the alkylation of the amino group with alkyl groups containing fluorine atoms making the most significant impact on optical data as seen by compounds **33**–**35** by shifting the absorbance values to 525–635 nm [44,47]. A study was also conducted where fluorinated chains were alkylated onto the amino groups (rhodamine F) to view the optical effects of increasing fluorine [45]. Compounds **44**–**46** were compared with increasing fluorine atoms. It is noticed that introducing more fluorine atoms to the rhodamine structure increases the absorption wavelength and molar absorptivity but decreases quantum yield while still reporting a comparably good quantum yield of 88–93%. The molar absorptivity values increased from 33,450 to 42,130 M*^−^*^1^ cm*^−^*^1^; although these values are also the lowest reported from the selected dyes. However, of the fluoroalkyl groups selected, fluoro-azetidine rings have the most significant effect on the rhodamine absorbances, compounds **33**–**35** showing the highest values of 525–635 nm and the greatest molar absorptivity values 122,000–167,000 M*^−^*^1^ cm*^−^*^1^.

Rhodamine B **37** and Si-rhodamines **35**, **48**, and **50** demonstrate the furthest redshift of the selected compounds. Although literature for fluorinated rhodamine B is scarce, compound **37** shows a significantly larger absorbance wavelength signal compared to other reported non-silicon rhodamines by more than 50 nm [25]; however, this is more likely due to the extension in the conjugation of the added group than the contribution of the fluorine that was added for functionality. Compounds **35**, **48**, and **50** show the most substantial differences in absorption and fluorescence wavelengths, mainly due to the Si-rhodamines being more redshifted than the typical oxygen-containing rhodamine scaffold; also, dyes **48** and **50** have the most significant absorbance values, 664 nm and 695 nm respectively, and this effect is likely attributed to the silicon atom alongside the fluorine atoms on the aromatic ring [47]. While these compounds absorb at 664 nm and 695 nm, respectively, it is essential to note the impact fluorine atoms and trifluoromethyl have on chemical structure and optical data. Compounds with the individual fluorine atoms bonded to the ring demonstrated signals at slightly larger wavelengths than trifluoromethyl, and the placement of fluorine atoms on the carbon adjacent to the amino groups of the scaffold further enhances this bathochromic shift.

### 3.2. Applications of Fluorinated Rhodamine

**Figure 9:**
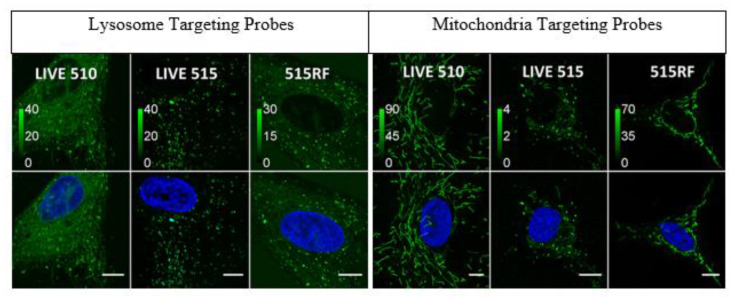
Targeting probes generated with rhodamine dyes **38**–**40** [44]. Confocal imaging of human fibroblasts with Hoechst 33,258 (blue) stain for cell nuclei and dyes **38**–**40** conjugated to pepstatin A for lysosome targeting (green) on the left and conjugated to (4-Carboxybutyl)-triphenylphosphonium for mitochondrial targeting (green) on the right; **38** is LIVE 510, **39** is LIVE 515, and **40** is 515RF in 1 μM solution. Scale bars: 10 μm. Figure used with permission from [44]. Copyright 2019 John Wiley & Sons.

Rhodamines are well-known dyes in the field of imaging. With new classes of rhodamine dyes emerging and new ways to shift fluorescence signals to higher wavelengths, it is crucial to recognize what substituents contribute the most innovation to the field. These compounds offer impressive imaging and selectivity for biomolecules to be applied as sensors for biological systems [43,44,49]. In Figure 9, the rhodamine-based targeting probes are synthesized using fluorinated rhodamine and different ligand attachments (Pepstatin A or (4-Carboxybutyl)triphenylphosphonium) to target the respective cellular organelles [44]. These fluorinated compounds exemplify bright fluorescence signals and improved organelle selectivity, which can be attributed to the enhanced lipophilicity from fluorine. From the images, dyes **39** and **40**, conjugated to Pepstatin A through an amide bond on the carboxy end, show the highest specificity for lysosomes and have low background signals. For imaging mitochondria, compounds **38** and **40** conjugated to (4-Carboxybutyl)triphenylphosphonium indicated the bright and specific imaging; however, compound **40** was noticeably phototoxic over time. Considering the varying degrees of fluorination, there is a biological preference for a limitation of fluorine atoms on the rhodamine scaffold, with few trifluoromethyl and fluorine atoms being ideal for biological applications of fluorophores.

**Figure 10:**
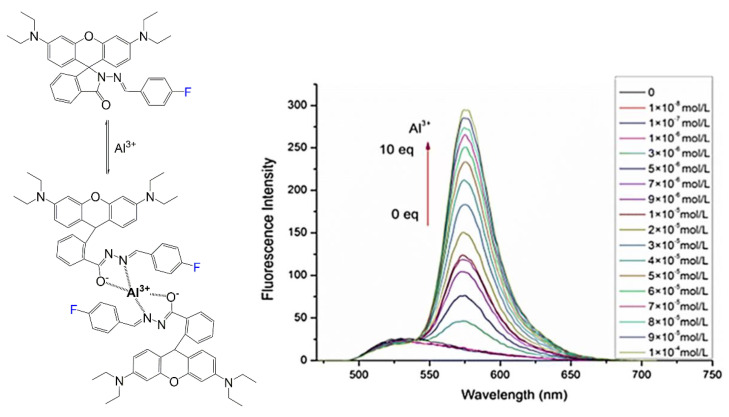
Metal sensing rhodamine dye **37**, selectivity for aluminum ions [43]. Binding mode in the presence of aluminum ion (**left**), and fluorescence emission spectra (**right**) at increasing concentrations of aluminum ion (0–10 equivalents) with 1 × 10^−5^ M of compound **37** in ethanol solution. Figure used with permission from [43]. Copyright 2019 Springer Nature.

Although rhodamine dyes are most notably recognized for their imaging capabilities, it is essential to consider other uses for rhodamines. Compound **37** is used as an aluminum ion sensor [43]. The study focuses on the different effects electron-withdrawing and electron-donating substituents contribute to functionality as a chemosensor. Although other versions can sense increasing metal ion concentrations, the fluorinated derivative had the most selectivity for metal ion aluminum over other metal ions where the fluorescence spectra are shown consistently increasing with increasing equivalents of aluminum ions (Figure 10). The left image shows the binding mode, showing the formation of a dimer when other studies predicted the stoichiometry of sensor **37** to aluminum to be 2:1 with oxygen negative charges and lone pairs of nitrogen interacting with the positively charged metal ion.

**Figure 11:**
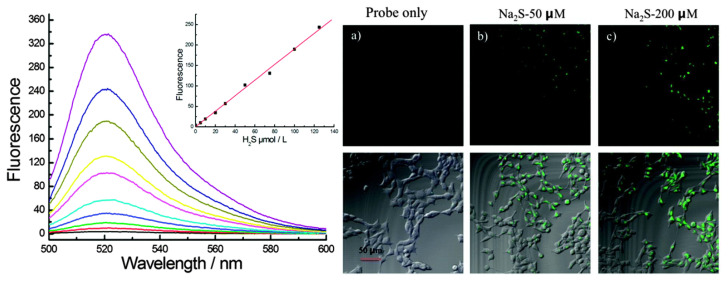
Biological sensing of hydrogen sulfide of dye **29** [41]. Fluorescence spectra (**left**) of 1 µM of dye **29** at concentrations 0–125 µM of hydrogen sulfide. Inset indicates a linear relationship between fluorescence and increasing concentrations of hydrogen sulfide. Confocal microscopy (**right**) of HEK293A cells incubated in 1 µM of dye **29** and two concentrations of sodium sulfide, 50 µM and 200 µM, respectively. Top images show fluorescence without the aid of a bight field. Scale bar: 50 µm. Figure used with permission from [41]. Copyright 2015 Royal Society of Chemistry.

In Figure 11, the reactivity of dye **29** for hydrogen sulfide is demonstrated; the fluorophore functions as a biological sensor with a turn-on mechanism with the increasing hydrogen sulfide presence [41]. The probe is reactive with the enzyme cystathionine β-synthase in cell studies. The confocal microscopy in Figure 11 shows the difference in the fluorescence signal seen in cells when sulfurous conditions are created and without. More imaging demonstrates the selectivity and sensitivity the probe has for hydrogen sulfide species and at increasing concentrations.

Along with these applications, rhodamine-based contrast agents are being designed for fluorescence signaling in the NIR region [24]. Rhodamines are known for their solubility and high quantum yields; what is needed are fluorophores with substituents that further improve optical properties or make the structure more biologically relevant. With this being considered, it is crucial to recognize the potential that rhodamine has for further biological imaging [50]. Literature shows potential for fluorinated rhodamine dyes being used for PET and NIR imaging [49,50].

## 4. Fluorinated Phthalocyanine Dyes

**Scheme 9:**
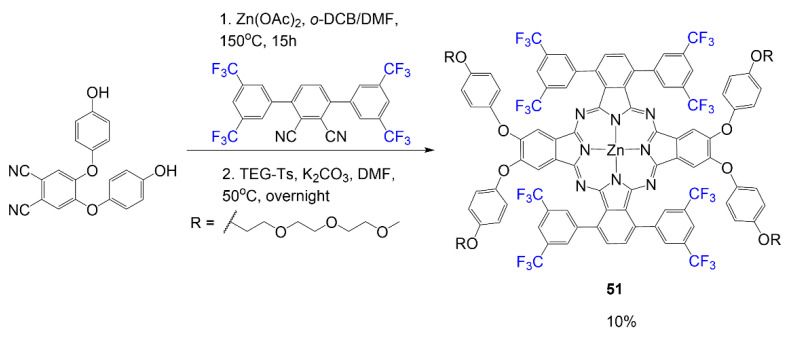
Synthesis of PEGylated fluorinated phthalocyanine [51].

In Scheme 9, the fluorinated phthalocyanine formation occurs in a cyclotramerization reaction requiring two phthalonitrile reagents [51]. In the first step, 4,5-bis(4′-hydroxyphenoxy) phthalonitrile reacts with 3,6-(3′,5′-bis(trifluoromethyl)-phenyl) phthalonitrile to form the phthalocyanine scaffold. The second reaction is the addition of the triethylene glycol (TEG) chains to the hydroxy ends of the phthalocyanine to render final product **51** in low yield 10%. The alkoxy groups are significant for the PEGylation step to improve the compound’s solubility for biological applications.

**Scheme 10:**
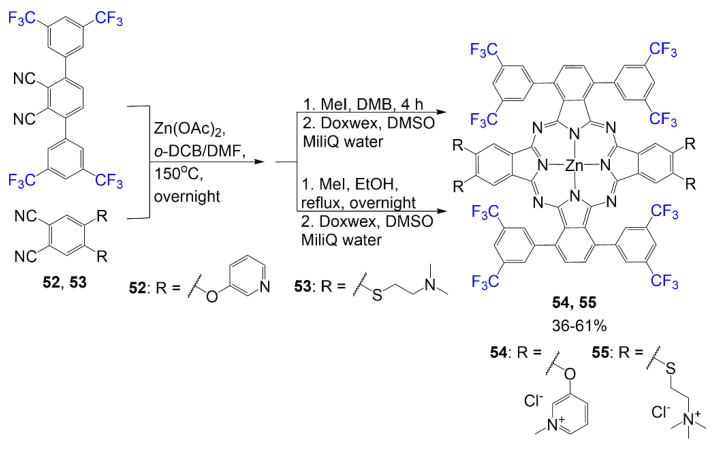
Synthesis of fluorine-containing tetracationic phthalocyanine dye [29].

The design of compounds **54** and **55** is also created with the intention of designing the compounds to overcome the hydrophobicity most phthalocyanines experience [29]. Scheme 10 begins with the cyclotetramerization reaction of phthalonitrile **52** or **53** with 3,6-(3′,5′-bis(trifluoromethyl)phenyl) phthalonitrile. In this attempt, quaternized nitrogen groups are introduced to the structure to promote hydrophilic properties to design an amphiphilic dye. These quaternized nitrogen-containing groups are introduced through an alkylated amine and alkylated pyridine. This alkylation is achieved using methyl iodide in the solvent ethanol to eventually generate compound **54** in 61% yield and compound **55** in 36% yield. These quaternized groups, alongside fluorine atoms, introduce favorable solubility for phthalocyanines due to the properties of quaternary salts.

**Scheme 11:**
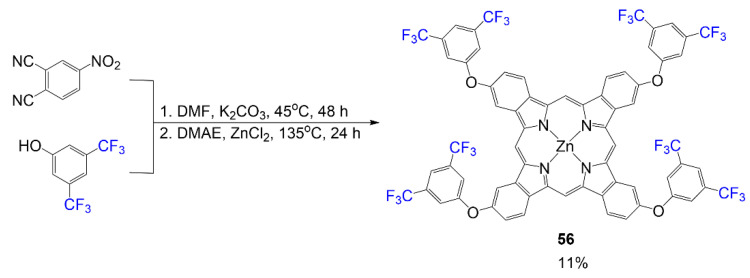
Synthesis of fluorinated phthalocyanine using 3,5-bis(trifluoromethyl)phenol [52].

Synthesis of the following phthalocyanine **56** in Scheme 11 begins with preparing the fluorinated phthalonitrile under basic conditions through a nucleophilic aromatic substitution reaction using 4-nitrophthalonitrile and 3,5-bis(trifluoromethyl)phenol mixture in DMF [52]. In step 2, a cyclotetramerization reaction occurred using dimethylethanolamine (DMAE) and zinc chloride to synthesize phthalocyanine **56** at a low yield of 11%.

**Scheme 12:**
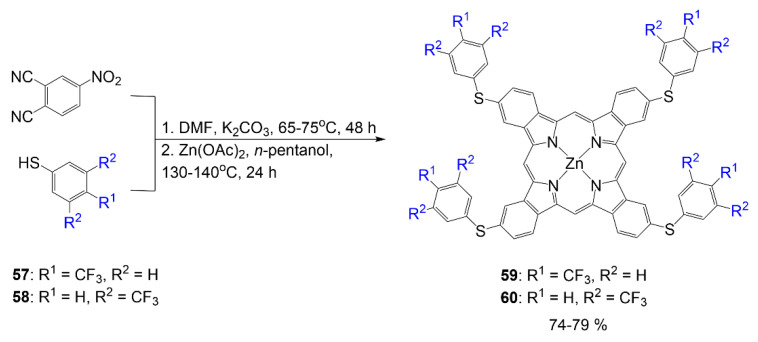
Synthesis of fluorinated phthalocyanine using 4-thiophenylphthalonitrile [53].

In addition, the synthesis of dyes **59** and **60** begins with the synthesis of 4-thiophenylphthalonitrile precursors that utilize 4-nitrophthalonitrile and thiophenols **57** and **58**, respectively (Scheme 12) [53]. Synthesis of the phthalonitrile precursor is achieved through normal phthalonitrile synthesis in DMF under basic conditions. In step two, zinc acetate is used as a metal source for the chelation in the formation of phthalocyanines **59** and **60** in a mixture and are isolated via column chromatography. The design of compound **60** is comparable to that of compound **56**, with the atom connecting the phenyl to the core phthalocyanine structure, which is sulfur rather than oxygen. The yield reported for compounds **59** and **60** falls within 74–79%.

**Scheme 13:**
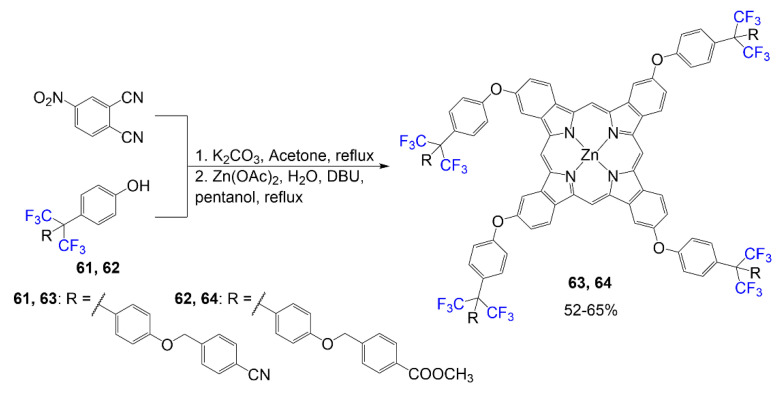
Synthesis of fluorophthalocyanine dyes **63** and **64** [54].

As shown in Scheme 13, relevant phthalonitriles are synthesized in potassium carbonate and acetone using 4-nitrophthalonitrile and corresponding phenols **61** and **62**, respectively [54]. The phthalocyanines **63** and **64** are formed from a reaction mixture of compound **61** or **62** in n-pentanol and DBU mix with Zn(OAc)·2H_2_O supplying the center chelating metal. The design of this compound is different due to the placement of trifluoromethyl groups on the sp^3^ carbon between two phenyl rings. The terminal ester and cyano groups were chosen for these structures to observe these phthalocyanines’ optical properties alongside the effects of fluorine. The yield reported for phthalocyanines **63** and **64** is 52–65%.

**Scheme 14:**
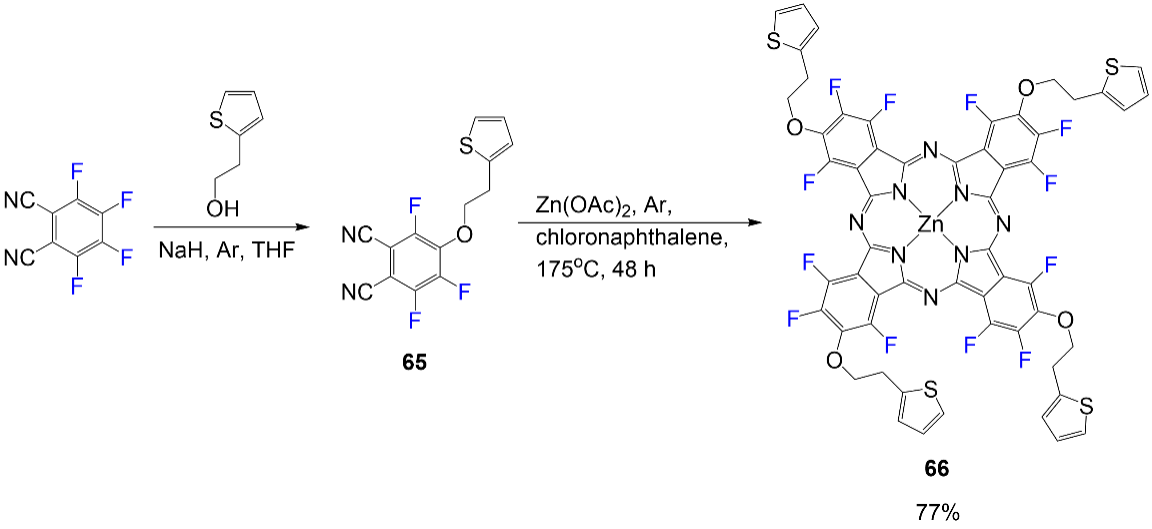
Synthesis of fluorophthalocyanine dye with thiophene arms [55].

The tetrafluorophthalonitrile reacts with 2-(2-thienyl)ethanol in inert conditions with a strong base to generating product **65** [55]. Once the desired phthalonitrile **65** is isolated, it is reacted in a mixture requiring four equivalents in typical phthalocyanine reaction conditions to yield the symmetrical phthalocyanine **66** at 77% (Scheme 14). Different variations of this phthalocyanine are derived from the different phthalonitriles synthesized.

**Scheme 15:**
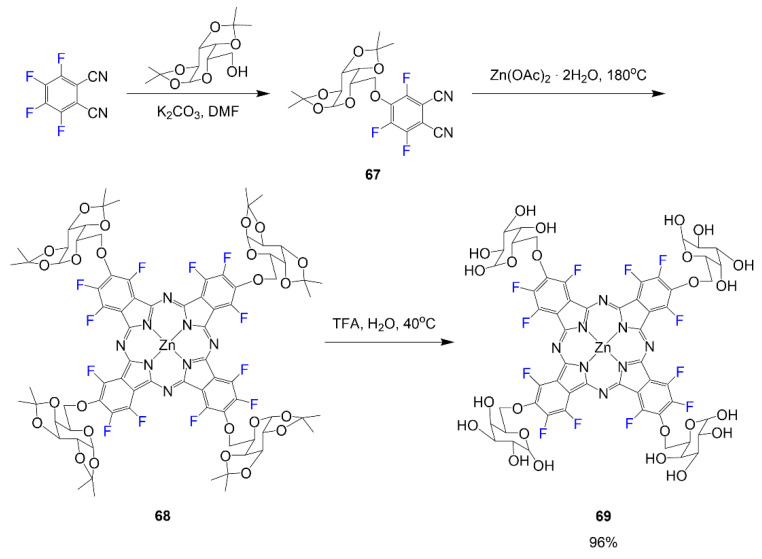
Fluorinated phthalocyanine containing galactose moieties [56].

In the work by Mori et al., sugar conjugated phthalocyanines were synthesized as outlined in Scheme 15 [56]. Like other phthalocyanines, a water-soluble group is conjugated to the phthalocyanine to improve solubility for the purpose of using the compound in further biological studies. In this molecule, 1,2,3,4-di-*O*-isopropylidene-α-d-galactopyranose is introduced to tetrafluorophthalonitrile to achieve synthesizing the desired phthalonitrile **67**. The tetrafluorophthalonitrile was used to synthesize the phthalocyanine **68** and was then treated with acid, Trifluoroacetic acid (TFA), to remove protecting groups to render final compound **69** with a yield of 96%.

### 4.1. Optical Properties of Fluorinated Phthalocyanine Dyes

Phthalocyanine dyes are compounds that typically fluoresce in the NIR optical window. This property is important to consider in these compounds due to their extensive conjugation system that makes them a vital class for contrast agents. However, some of the most significant problems with these compounds are their solubility and quantum yield. In most of the previously described classes of fluorophores, the compounds typically demonstrated higher quantum yields and molar absorptivity corresponding to the number of fluorine atoms introduced to the structural design.

In Table 3, optical data is reported for the selected compounds shown. All the described compounds show absorbance data at or above 650 nm, which is typical for phthalocyanine. The compounds notice the highest absorbance values with the fluorinated phenyl groups coming off the aromatic corners for the phthalocyanine core, such as compounds **51**, **55**, and **60** showing absorbances of 712 nm, 694 nm, and 695 nm, respectively. Also, fluorinated compounds containing sulfur such as **55**, **60**, and **66** show greater absorbance values as well.

However, it is necessary to note the effects viewed in compounds **59** and **60** since they have different amounts of fluorine atoms. Compound **60** has two trifluoromethyl groups on each of the branching rings, while compound **59** has one. The absorbance values also correspond in this structural design, with compound **60** having a larger absorbance value by 18 nm compared to dye **59**. In literature, a similar phthalocyanine was reported by Çelenk Kaya et al. with the structure different in one carbon between sulfur and the phenyl ring, indicating a similar absorbance of 694 nm compared to the absorbance of 60 [57]. Fluorophore **60** is also similar to the structure of compound **56** with how many fluorine atoms are present in the chemical structure and differing sulfur for oxygen; however, the absorbance of dye **56** is similar to the value of compound **59**, demonstrating the effect of the trifluoromethyl group on the aromatic ring being comparable to the effect sulfur has on phthalocyanine.

Similarly, it is essential to note the 16 nm difference in absorbance wavelength between dyes **55** and **54** differing in oxygen versus sulfur bonding of moiety to the scaffold. Although this atom difference is important to recognize, it is also necessary to consider the difference in solubility between the two compounds. Compound **54** having a 134,900 M*^−^*^1^ cm*^−^*^1^ molar absorptivity versus **55** having 158,500 M*^−^*^1^ cm*^−^*^1^. Although, this value is not surprising considering dye **54** has a pyridine moiety contributing to the already planar structure of the phthalocyanine scaffold, while dye **55** has a rotatable trimethylammonium arm. In terms of solubility, dyes **63** and **64** had the most significant molar absorptivity values above 200,000 M*^−^*^1^ cm*^−^*^1^, which is likely due to its large moieties to combat aggregation. However, compounds **68** and **69** utilized sugar moieties to improve solubility, but molar absorptivity values were not reported to compare optical data on this property.

It is mentioned in several articles that the aggregation of phthalocyanines diminishes the potential of these compounds to be used as imaging agents. The aggregation will diminish fluorescence signals leading to low quantum yield and is also associated with poor solubility. Some of the most necessary modifications reported are the use of bulky fluorine-containing functional groups [58,59,60] to combat aggregation using steric hindrance and improved solubility.

### 4.2. Applications of Fluorinated Phthalocyanine Dyes

One of the prime examples of the application of phthalocyanine dyes is in PDT studies due to their ability to function as photosensitizers that follow a mechanism for the generation of singlet oxygen species [61]. These PDT studies have been done most recently on various cancer and bacterial cells. Fluorinated phthalocyanines have also shown to be relevant chemical sensors being used for the detection of gases, most notably ammonia [55,62,63,64]. It has also shown recent applications as a chemical sensor used to detect nitrogen dioxide [65,66]. Fluorinated phthalocyanines are typically compared to their nonfluorinated versions to observe the degree of improvement in properties; in another study, fluorinated phthalocyanines were tested as organic semiconductors [67].

**Figure 12:**
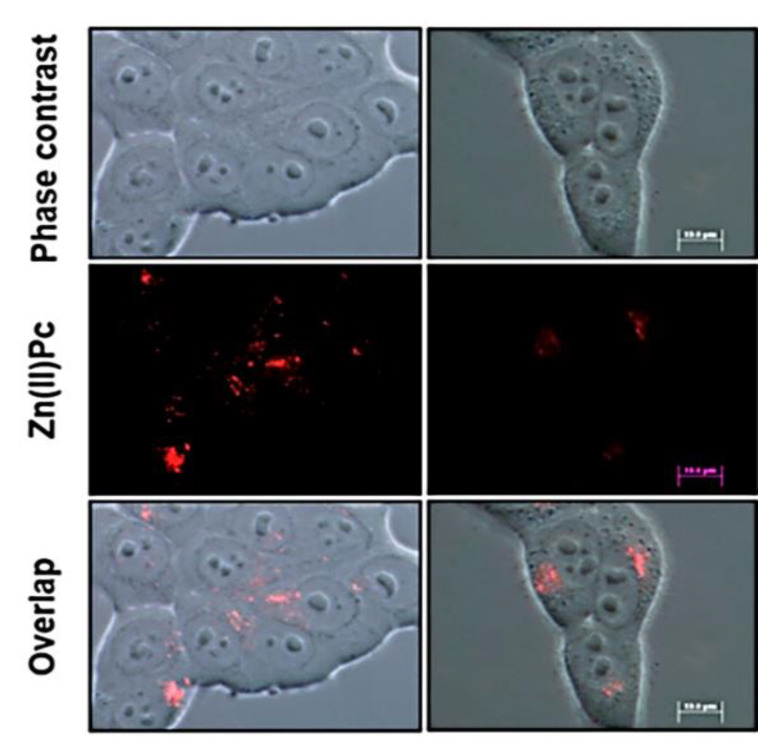
Cellular localization study of compound **51** in SCC-13 (**left**) and HeLa cells (**right**). Fluorescence microscopy conducted with green-light irradiation at 545 nm using a BP545 filter. Figure used with permission from [51]. Copyright 2020 Revuelta et al.

Compound **51** from Scheme 9 utilizes aryl groups containing two trifluoromethyl substituents as bulky groups to combat aggregation and improve solubility alongside PEG chains [51,68]. Although these changes improve optical properties, cell imaging for this compound is not considered as desirable as other agents. In Figure 12, compound **51** demonstrates a red signal within the cell structure, indicating some localization in organelles; however, the signal is too weak but shows the potential for phthalocyanine improvements using similar structural modifications.

**Figure 13:**
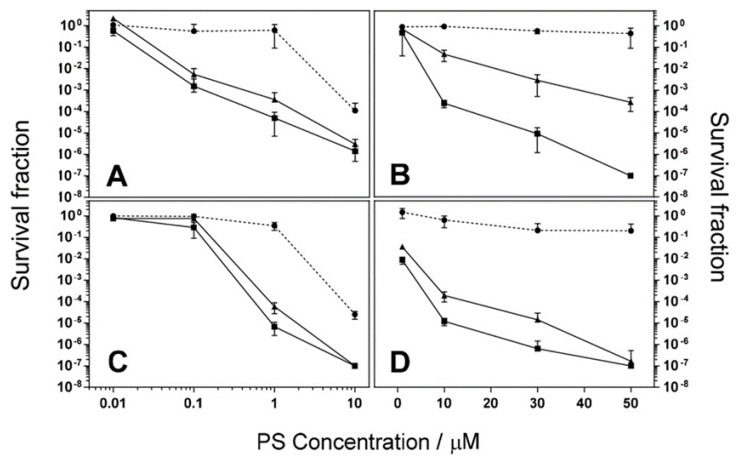
Bacterial survival of S. aureus (**A**,**C**) and E. coli (**B**,**D**) after photodynamic inactivation treatment. Compound **54** effects viewed in (**A**,**B**), and compound **55** viewed in (**C**,**D**). Circles represent dark toxicity (J·cm^−2^), triangles (J·cm^−2^), and squares (J·cm^−2^). Figure used with permission from [29]. Copyright 2020 Elsevier.

Phthalocyanines have promising data as photosensitizers for PDT studies. In Figure 13, the observed data indicates the potential of phthalocyanines being used for the inactivation of bacteria [29]. Like the structure of compound **51**, the phthalocyanines showed that **54** and **55** demonstrate bulky bis(trifluoromethylphenyl) in two positions of the phthalocyanine and other water-soluble substituents applied to the phthalocyanine core. In this example, the water-soluble substituents are pyridinium for compound **54** and alkylammonium for compound **55**. Figure 13 shows the photochemical activity of compounds **54** and **55** against the bacteria, E. coli and S. aureus upon introducing red-light irradiation by indicating decreasing survival as dye concentrations increase.

**Figure 14:**
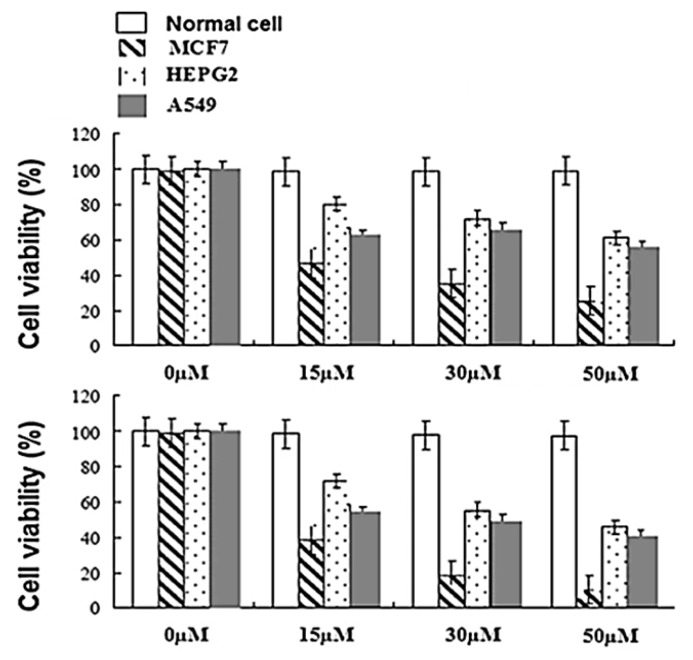
Cell viability for compound **59** (**top**) and compound **60** (**bottom**). Cells analyzed by MTT assay and treated with different concentrations (15, 30, and 50 µM) of dyes **59** and **60**. Figure used with permission from [53]. Copyright 2018 Springer Nature.

Many PDT studies of phthalocyanine activity are in cancer cell studies. Phthalocyanines of different structures have proven to be useful anticancer agents [51,53,63,68,69]. Figure 14 compares two zinc phthalocyanines with differing degrees of fluorination incorporated into the structure; compound **59** contains four trifluoromethyl groups, and compound **60** contains eight trifluoromethyl groups. Based on the presented data in Figure 14, it is notable that compound **60** trifluoromethyl groups are more effective against the selected cancer cells than dye **59**; however, both compounds are more useful for PDT than the nonfluorinated version of the molecule. The data is quite reasonable, with minimal effects seen on normal cells while decreasing cell viability with increasing concentrations of the fluorophore.

**Figure 15:**
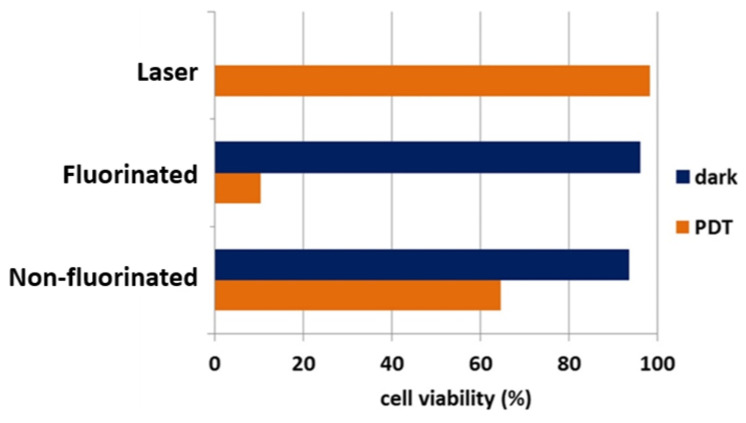
Effect of fluorine on galactose-containing phthalocyanine photosensitizer **69**. Cell studied are HT-1080 cells in red light irradiation (664 nm) and dark conditions (without laser). Figure used with permission from [56]. Copyright 2015 Elsevier.

In compounds **68** and **69**, as outlined in Scheme 15, peripheral galactopyranosyl moieties were used on a fluorine-containing phthalocyanine; **68** is the version with protecting groups on the galactopyranosyl while dye **69** is the unprotected version being studied for PDT [56]. Synthesized along with these compounds were the nonfluorinated version of the compounds to compare the effect fluorine has in the PDT studies. Figure 15 shows that the fluorinated agent **69** is more effective against HT-1080 cells than the similar nonfluorinated version. The protected compound **68** versus unprotected compound **69** were also studied and compared to each other. It was observed that the protected version was ineffective by showing cytotoxicity in the dark and after laser. These results highlight the importance of the hydroxy groups in the peripheral galactopyranosyl of dye **69** and fluorine atoms for amphiphilicity of the phthalocyanine.

## 5. Fluorinated Cyanine Dyes

**Scheme 16:**
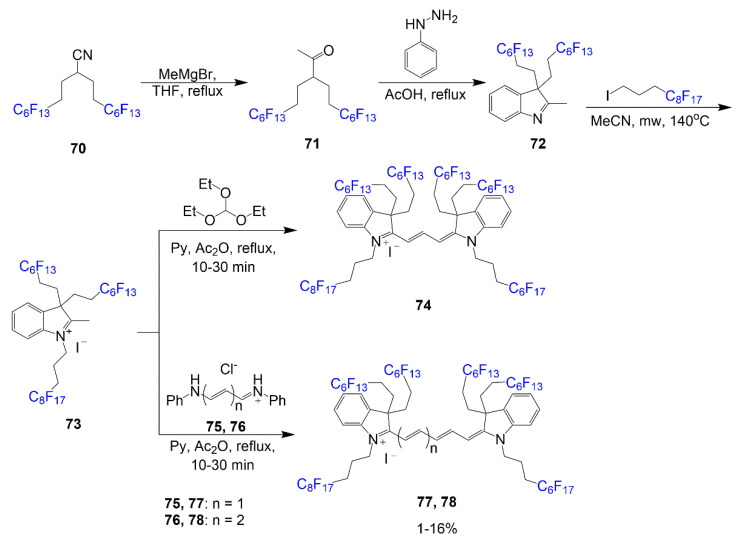
Synthesis of cyanine dyes with perfluorocarbon chains [70].

As seen in Scheme 16, cyanine dyes with perfluorocarbon chains were synthesized; these compounds were designed with the intention of being studied for nano/microdroplet studied in biological systems [70]. The fluorinated chains modify the compound to be soluble in the fluorous phase opening for application avenues. The synthesis outlined in Scheme 16 demonstrates a three-step process for the creation of the indolium **73**. To begin, the nitrile **70** reacts with Grignard reagent to render a ketone **71**. This ketone is reacted with phenylhydrazine to form the ring using the same conditions typically used to form heterocycles of this kind. The indoline **72** is alkylated with another fluorous reagent to form the indolium salt **73**. This salt reacts with the corresponding bridge forming reagent, triethyl orthoformate, reagent **75**, or reagent **76**, for the final step to create final dyes **74**, **77**, and **78** respectively in low yields within 1–16%.

**Scheme 17:**
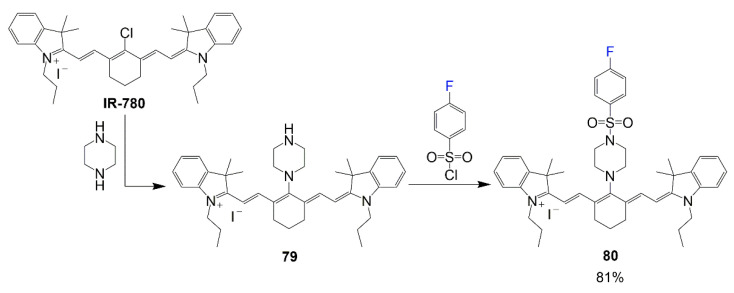
Synthesis of lysosome targeting NIR probe [33].

The synthesis shown in Scheme 17 demonstrates the use of IR-780 modified into becoming a bioprobe specified for lysosome targeting [33]. IR-780 reacts with piperazine to substitute the chlorine for the nitrogen of piperazine to create intermediate **79** via S_NR1_ reaction. This piperazine functions as a bridge to aid the connection of the p-fluorobenzenesulfonyl group to the dye bridge, as seen in product **80** with a good yield of 81%.

**Scheme 18:**
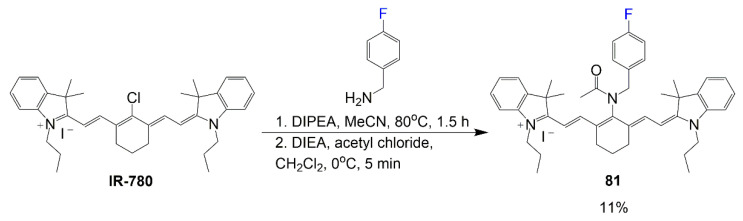
Synthesis of cell-targeting fluorinated fluorophore [32].

Similar to Scheme 17, the synthesis of heptamethine dye **81** begins with IR-780 being modified for specified targeting (Scheme 18). However, this molecule design is studied further to pinpoint relevant cells targeted as well as the specified enzyme, HMOX2, interacting most effectively with the fluorophore [32]. The reaction in Scheme 18 is two steps, with the first one considering the reaction with 4-fluorobenzylamine, IR-780, and DIPEA in basic conditions to substitute the chlorine for the primary amine. The second step considers the introduction of a acetyl chloride and *N*,*N*-diisopropylethyl amine (DIEA) for the amide bond formation. Although applications of this compound are quite promising, the synthetic yield of final compound **81** is a low 11% after the two steps.

In the synthesis shown in Equation (4) by Zheng et al., fluorine-containing asymmetric hemicyanine dyes are designed using xanthene at one end and indolium at the other end [71]. These compounds begin with the same hemicyanine intermediate **82** and undergo a substitution reaction with different nucleophiles to afford fluorinated dyes **83**–**89** in yields varying between 16% and 65% based on the nucleophile used.


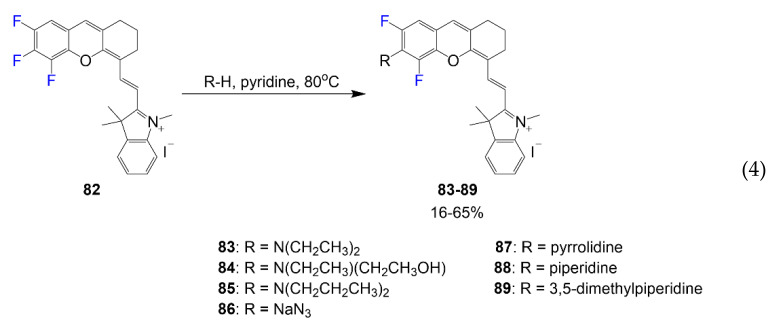


The reaction in Equation (5) utilizes the benz[c,d]indolenine aldehyde intermediate **90** or **91** with water-soluble heterocyclic salt **92** in acetic anhydride under reflux for dye synthesis [72]. The aldehyde arm of compound **91** or **92** is intended to afford the dye bridge for the final compounds **93** and **94**. This reaction affords the final product at a 49% yield for **93** and 18% yield for **94**, as reported earlier by the Henary group [72].


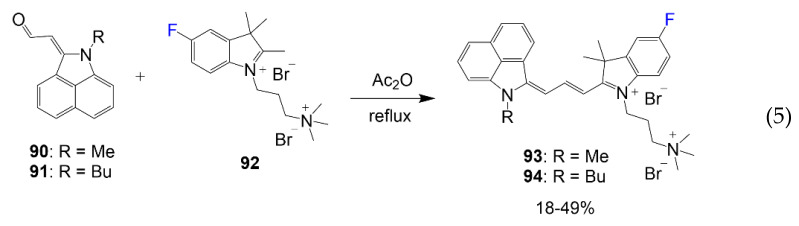


**Scheme 19:**
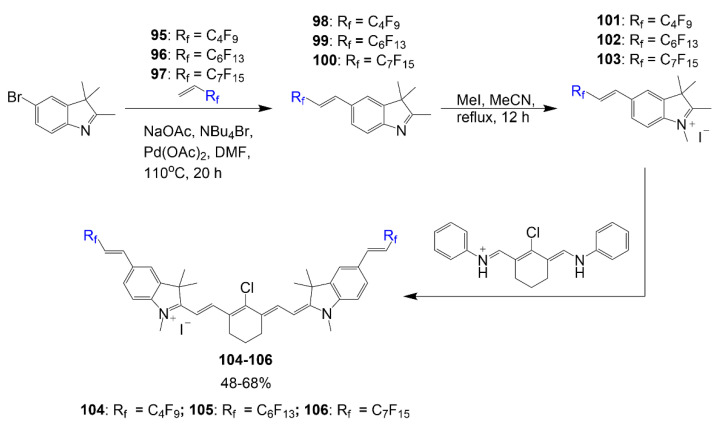
Synthesis of polyfluorinated cyanine dyes [73].

The synthesis of polyfluorinated cyanine dyes designed by Braun et al. begins with a bromine-containing heterocycle reacting with compounds **95**–**97** to generate the fluorinated heterocycles necessary in Scheme 19 [73]. These polyfluorinated heterocycles **98**–**100** are then alkylated with methyl iodide to render indolium salts **101**–**103**. The respective salts, **101**–**103**, react with a known linker reagent to form the corresponding dyes **104**–**106** in good yields within 48–68%. The polyfluorinated chains in the reaction experience enhancements in drug properties leading them to be considered selective for mitochondria targeting.

**Scheme 20:**
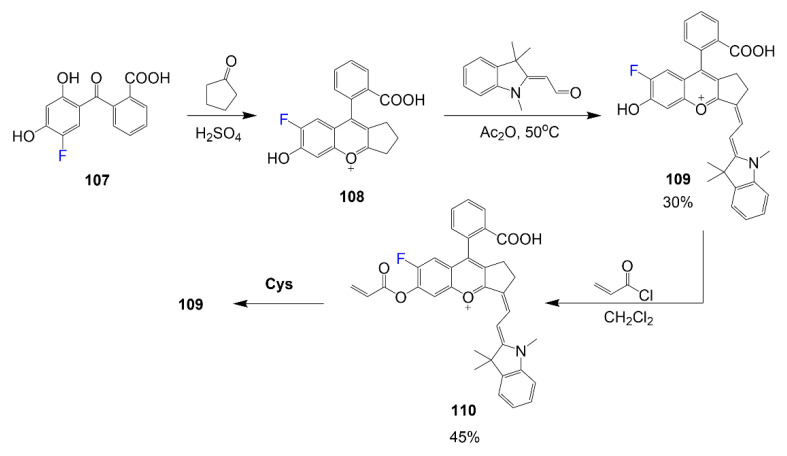
Synthesis of fluorine-containing chromenylium-cyanine [74].

Another synthesis for fluorine containing cyanine dyes is shown in Scheme 20, which begins with the reaction of carboxylic acid derivative **107** and cyclopentanone under acidic conditions. The second step shows the formation of the fluorinated heterocycle **108** reacting with indolium-containing half dye to form chromenylium-cyanine **109** in acetic anhydride [74]. The free hydroxyl group of compound **109** then reacts with acryloyl chloride in dichloromethane to form fluorescently active **110**. In the presence of cysteine residues, the active dye can be reverted to nonfluorescent compound **109**.

**Scheme 21:**
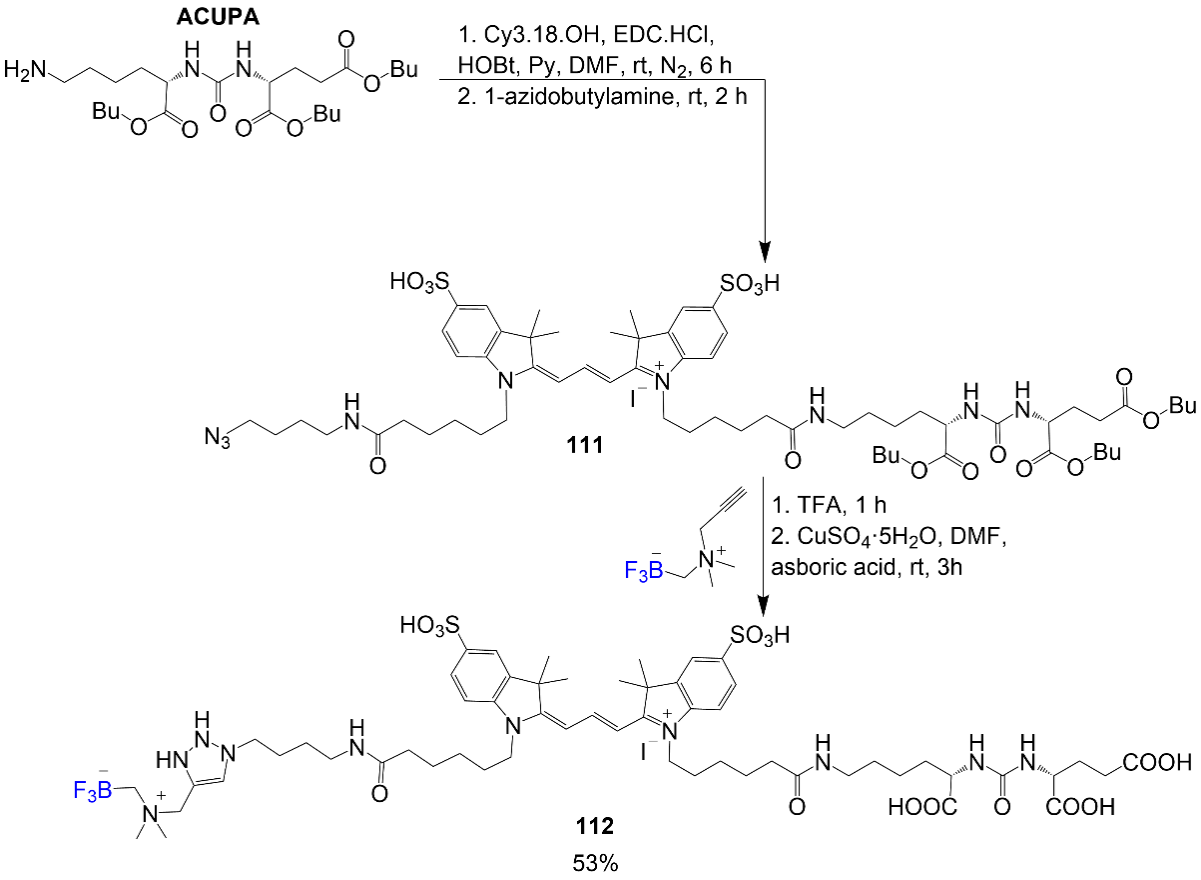
Synthesis of trimethine dye for 18F-PET of prostate cancer [75].

Considering the importance of imaging, a PET active trimethine dye is shown in Scheme 21 and is achieved in four steps starting from a derivative of Cy3 that reacts with ACUPA, and a step after, 1-azidobutylamine is introduced to one of the amide bonds, thus forming organic dye **111** [75]. The following two steps consider acidification to deprotect the carboxylic acid groups in compound **111** and condense the alkylammoniomethyl trifluoroborate by forming the ring at the azide, forming final compound **112** with a yield of 53%. The fluorine introduced to the structure will be functionalized upon a radiolabeling procedure for PET imaging.

**Scheme 22:**
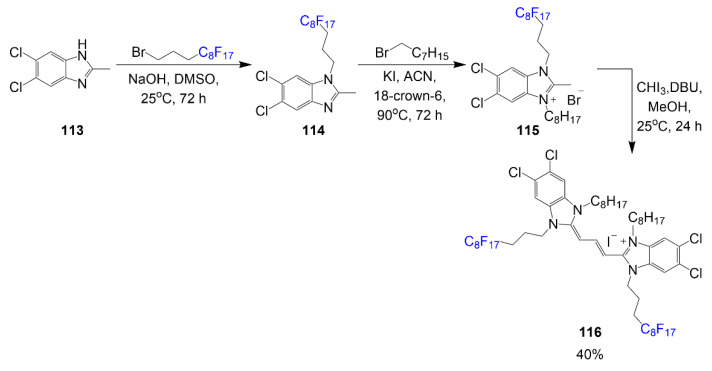
Synthesis of fluorous cyanine dye for J-aggregation [76].

Fluorinated dye by Cao and Sletten was synthesized to observe J-aggregation in fluorous solvent as shown in Scheme 22 [76]. Synthesis begins with the alkylation reactions between compound **113** and 1-bromo-3-(perfluorooctyl)propane under basic conditions to generate heterocycle **114**. The second step requires the alkylation of the opposite nitrogen with 1-bromooctane to afford salt **115**. Two equivalents of salt **115** with iodoform and DBU in a reaction mixture produce final product **116** with a good yield of 40%. Optical properties of this dye were observed in aqueous and fluorous (perfluorocarbons) media to compare the enhancing effects of fluorine. Synthesis of the cyanine was completed in a three-step process with two alkylation steps (one on each nitrogen), and the third step is the dye formation.

**Scheme 23:**
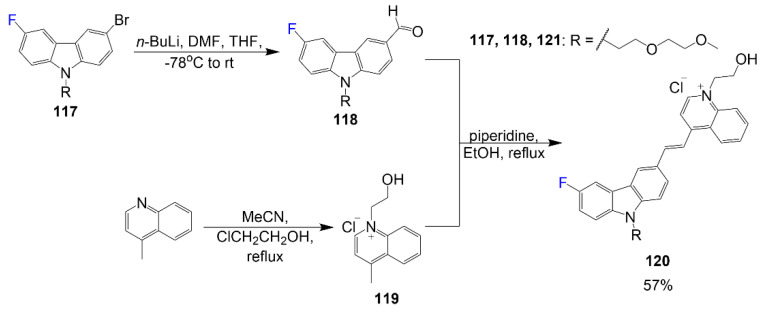
Synthesis of fluorinated cyanine dye for labeling of amyloid-β oligomers [77].

In addition, Scheme 23 begins with the reaction of compound **117** with n-butyllithium in a THF/DMF solvent mixture to obtain compound **118** [77]. To synthesize salt **119**, 4-methylquinoline reacts with 2-chloroethanol in acetonitrile. A reaction mixture of compounds **118** and **119** in piperidine under reflux synthesizes the final compound **120** with 57% yield. Fluorophore **120** is designed with an ethylene glycol group to improve solubility in aqueous solvent and a hydroxy and fluorine moiety for improvement of intermolecular interactions relevant for good binding affinity and amyloid-β oligomer selectivity.

**Scheme 24:**
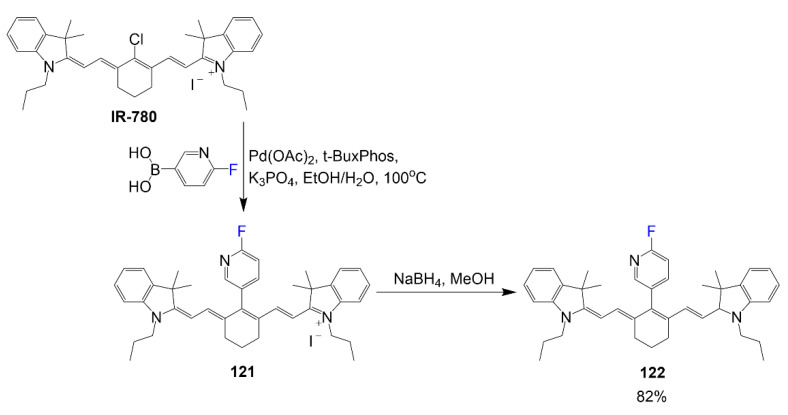
Fluorine-containing hydrocyanine for the generation of ROS [78].

The hydrocyanine **122** reported by Al-Karmi et al. shows the synthesis of a multimodal cyanine probe [78]. As outlined in Scheme 24, the synthesis of compound **122** begins similar to compounds **80** and **81** with the IR-780 as the precursor for further modifications. Upon substitution of the chlorine atom with the 6-fluoropyridin-3-ylboronic acid, the active compound **121** is formed. This intermediate undergoes a reduction step to generate the nonfluorescent hydrocyanine **122** in 82% yield. The hydrocyanine described is designed to be a multimodal “turn-on” optical probe for reactive oxygen species sensing and PET active through ^18^F labeling. It is also important to notice that there is a fluorine atom on the aromatic ring similar to compounds **80** and **81**, indicating a trend in this type of structure for specific biological targeting.

Henary Group reported the synthesis of halogen-containing pentamethine dyes reported [79]. From the series of these halogenated dyes, the fluorine-containing dyes are reported in Equation (6). As outlined, dyes **128**–**133** are synthesized from different combinations between respective salts **123** or **124** in a reaction mixture with corresponding linkers **125**–**127** in basic conditions; the yield of this dye reaction is 37–78%. These dyes were designed to study the effects electron-withdrawing moieties have with halogen-containing linkers to observe their structure-inherent targeting in biodistribution studies.


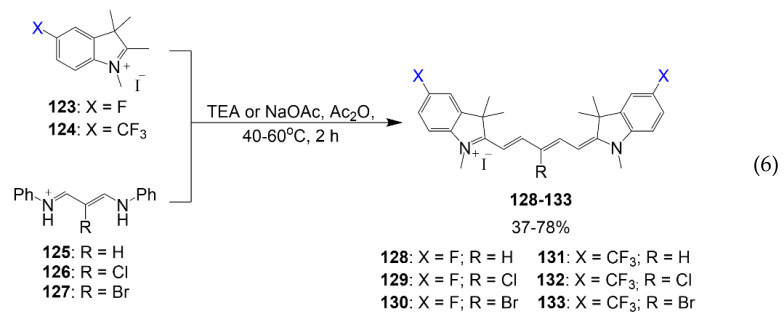


### 5.1. Optical Properties of Fluorinated Cyanine Dyes

The optical properties of the selected cyanine dyes, as seen in Table 4, show that extension in dye conjugation had the most significant impact on red-shifting modified dyes. It is shown that many of the selected cyanine dyes report low quantum yields while some are unreported. Since there are not so many reports of dyes with varying degrees of fluorophilicity, more studies are recommended to confirm the effect fluorine has on this class fluorophore optically. Although not all reported, the solubility of the reported cyanine in polar solvents is relatively high based on the molar absorptivity.

To begin, it is crucial to address the dyes from Table 4 with perfluorocarbon chains. Compounds **104**–**106** have the greatest absorbance values of **803**–**805** nm, which can be attributed to the alkene extending the conjugation of the aromatic carbon of the indolines in the structure. Although compounds **104**–**106** have varying degrees of fluorine, their optical properties are quite similar with compound **105**, specifically with compound **105** being better than compounds **104** and **106** in molar absorptivity 256,300 M*^−^*^1^ cm*^−^*^1^ and Stokes shift of 17 nm. Other compounds with perfluorocarbons, such as compounds **77** and **78**, have high higher molar absorptivity values; dyes **77** and **78** have relatively high quantum yields compared to some of the compounds listed with quantum yields of 16–29%. Absorbance/emission wavelength and quantum yield of dye **116** are considerably low at 7% compared to other listed cyanine dyes, and trimethine dye **74** optical data is low compared to pentamethine **77** and heptamethine **78** versions of the dye.

Cyanine dyes have versatile optical properties based on the functional groups introduced to them. In recent literature, a few cyanine dyes had aromatic fluorine exposed from the linker chain, as shown in dyes **80**, **81**, **121**, and **122** [32,33,78]. Dyes **121** and **122** are similar structures with the difference of one double bond differentiating between the fluorescent **122** versus the nonfluorescent **121** structure. Compounds **80**, **81**, and **122** have different degrees of conjugation. Primarily, compound **80** has a break in conjugation between the dye bridge and the phenyl ring. Dye **81** and **122** have more extended conjugation from the dye bridge than dye **80**, but compound **81** has more possible resonance structures due to the amide bond, which would support the observation of the dye having the greatest absorbance and emission wavelengths of 805 nm and 825 nm. Although the fluorine atoms introduced in these structures are not the primary contributors to their optical data, the fluorine atom contributes to the functionality of the dye as a probe. Although dye **112** is structurally different from the dyes with aromatic fluorine atoms, it is also highly recognized for its functionalities versus improvements to the scaffold optically.

Chromenylium-cyanines **109** and **110** and other hemicyanine dyes selected show low quantum yields and with some compounds on the blueshifted end of the cyanine group. Hemicyanine dyes **83**–**89** focus more on the positions of the fluorine atoms aiding in nucleophilic substitution rather than improvements in optical properties, which is notable based on the low quantum yields 1–5% [71]. Dyes **93** and **94** offer limited details about optical data, but asymmetric dyes of this type would be expected with absorbance above 700 nm. Optical data for compound **120** is quite unpredictable with such a large Stokes shift and low solubility based on the functional groups observed; however, the fluorine is more effective for membrane penetrability and low bio-toxicity.

Cyanine Dyes **128**–**130** with fluorine atoms have better molar absorptivity values (above 200,000 M*^−^*^1^ cm*^−^*^1^) than dyes **131**–**133** with trifluoromethyl groups; however, dye **131** has the best quantum yield out of the series by almost more than double with a value of 59%. The fluorinated cyanine dyes in this series also have slightly greater Stokes shifts than the version of these dyes with hydrogen in the place of fluorine.

### 5.2. Applications of Fluorinated Cyanine Dyes

**Figure 16:**
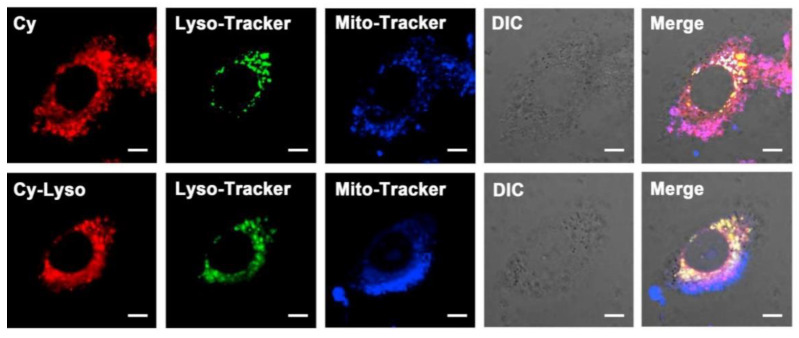
Confocal microscopy of **IR-780** (Cy; top) and dye **80** (Cy-Lyso; bottom) in A549 cells [33]. Red staining shows probe **80** excited at 639 nm. Green staining shows Lyso-Tracker (λ_ex_ = 555 nm). Blue staining shows Mito-Tracker (λ_ex_ = 488 nm). Differential interference contrast (DIC) microscopy and merged images. Concentrations of 1 μM for each. Figure used with permission from [33]. Copyright 2020 Elsevier.

Cyanine dyes are primarily known for their applications in fluorescence imaging. Like other compounds, they can be modified for different functionalities. Figure 16 shows the confocal microscopy done to prove the lysosome targeting ability of compound **80**; structure seen in Scheme 17. Abbreviation Cy represents IR-780 in the images [33]. IR-780 shows a signal for lysosome and mitochondria as expected of a typical cyanine dye. Dye **80** is specified for lysosomes and shows aggregation in the region of the cell where lysosomes are more concentrated as opposed to around the entirety of the cell-like Mito-Tracker. Differential interference contrast microscopy shows the outline of the cell, while the merged images show the overlap.

**Figure 17:**
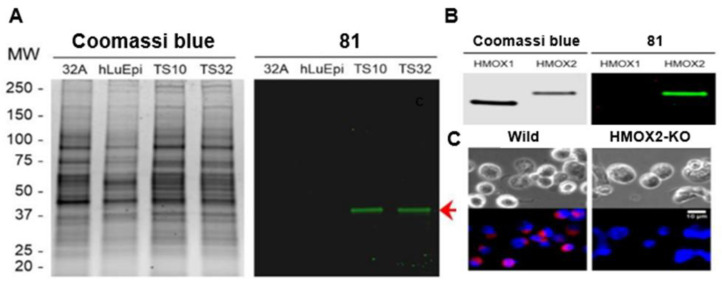
Target identification of dye **81** and therapeutic effects. (**A**) SDS-PAGE identified dye **81** in TS10 and TS32 cells. (**B**) SDS-PAGE analysis indicated the reaction of dye **81** with HMOX2. (**C**) Cell fluorescence images between wild type and HMOX2-KO cells. Figure used with permission from [32]. Copyright 2019 American Chemical Society.

Cyanine dyes are advantageously used for targeted imaging. They are highly regarded for their abilities to label biomolecules, and many studies focus on making modifications to their heterocyclic ends and or modifications on the linker bridge. Hydrocyanines are nonfluorescent tunable dyes that will convert to their fluorescent cyanine version in the presence of reactive oxygen species [78,80]. As presented in Scheme 24, dye **122** is prepared with 6-fluoropyridin-3-ylboronic acid as a stable prosthetic group that can undergo further radiolabeling for PET functionality [32]. In Figure 17, a study is shown about the targeting abilities of one dye **81** out of a library of compounds. In the Figure, affected cells are isolated (Figure 17A), enzyme specificity is tested (Figure 17B), and specificity for the enzyme is observed (Figure 17C). From this data, it is concluded that compound **81** works as a therapeutic agent that affects the functions of HMOX2 to block tumor growth. Like compounds **80** and **122**, compound **81** shows one of the most significant contributions fluorine plays in improving dye targeting abilities.

**Figure 18:**
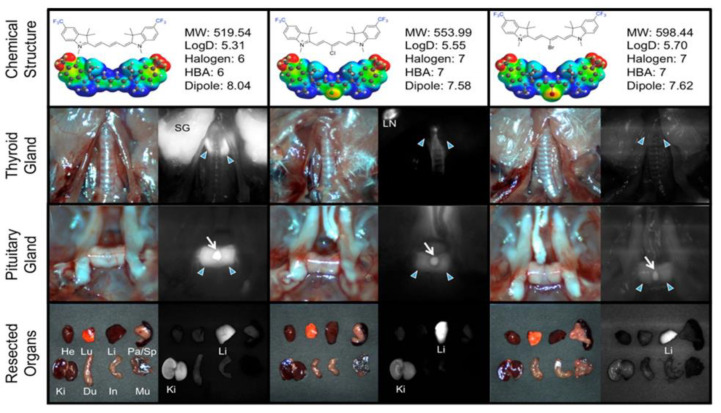
Biodistribution studies for compounds **131**–**133** indicating thyroid and pituitary gland targeting. Each mouse model was injected with 10 mM of the respective fluorophores: **131** (**left**), **132** (**middle**), and **133** (**right**). Arrow heads indicate the anterior portion and arrows for posterior pituitary. Figure used with permission from [79]. Copyright 2016 American Chemical Society.

Many research groups generate a library of compounds with different modifications to study the differences certain functional groups and moieties contribute to therapeutic abilities or cell/organ targeting [79]. Some of the smallest changes have significant impacts on which organs are targeted and what degree of signal strength is observed. In the biodistribution study shown in Figure 18, the electronic contributions trifluoromethyl groups offer to the cyanine scaffold and the effects a halogen on the dye bridge exhibit on the fluorescence signal are presented. The dye exhibits an excellent signal in the pituitary gland (Figure 18). It is also noted the high signals of other endocrine tissues, which are observed in the version of the dye without halogen in the dye bridge.

## 6. Conclusions

In the past decade, fluorine has been incorporated into many fluorophores in an attempt to improve chemical and optical properties. Several classes of fluorophores have creatively applied some of the same fluorinating trends to add different applications to some well-known scaffolds that have the potential to be modified to give redshifted absorbance values. Halogenation of molecules has shown to have positive effects on the therapeutic properties of many of these scaffolds, taking into consideration the size and electronegative properties of fluorine. It is interesting to view the chemical design that many research groups were able to achieve as well as some of the significant applications of their dyes from fluorescence imaging, chemical sensing, and adding PET imaging functionality. In some other compounds, fluorine or trifluoromethyl groups improved the synthetic efficiency, improved solubility, and combat aggregation of planar scaffolds. This review highlights a trend in the use of perfluoroalkyl chains, trifluoromethyl, and varying degrees of aromatic fluorine atoms in different scaffolds. As many modifications have been explored in various scaffolds, it is essential to explore further some modifications that have worked well in certain scaffolds and apply them to others.

The scaffolds chosen and detailed in Figure 2 indicate the most popular scaffolds where fluorine atoms have been modified onto the structure. There are other scaffolds that have been reported recently; however, these types of fluorine modifications have not been explored as heavily. It is important that that scientific community further explores these fluorination techniques in other dye scaffolds; it is also necessary that the dyes currently designed be applied to further studies to fully understand the optical properties and biological applications of these fluorinated scaffolds. Many compounds emphasized the new synthesis methods while offering opportunities for biological applications without providing data in this area, which would be beneficial to the scientific community.

## Figures and Tables

**Table 1:** Optical properties of selected fluorine containing BODIPY dyes.

Dye	λ_Abs_ (nm)	λ_Em_ (nm)	ε (M^−1^ cm^−1^)	Stokes Shift (nm)	Φ_F_	Solvent	Applications
**2**	531	550	103,000	21	0.05	THF	^19^F MRI
**3**	645	657	99,000	12	0.04	THF	^19^F MRI
**4**	531	551	138,000	20	0.04	THF	^19^F MRI
**5**	640	653	104,000	13	0.27	THF	^19^F MRI
**9**	502	509	102,000	7	0.95	Cyclohexane	Optical **
**10**	525	541	78,000	16	0.94	Cyclohexane	Optical **
**11**	532	571	54,000	39	0.76	Cyclohexane	Optical **
**16**	735	780	59,815	45	0.42	DMSO	^19^F MRI/NIR/PA
**21**	556	587	46,600	31	0.91	Acetonitrile	Optical **
**22**	550	587	58,100	37	0.94	Acetonitrile	Optical **
**24**	528	541	56,500	13	0.72	Acetonitrile	NIR *
**25**	637	650	70,500	13	0.80	Acetonitrile	NIR *
**26**	501	513	---	12	---	DCM	^19^F MRI
**27**	580	590	---	10	---	MeOH	PET

* NIR used for fluorescence imaging compounds*;* ** Optical used for compounds used for fluorescence studies in visible range.

**Table 2:** Optical properties of selected fluorinated rhodamine dyes.

Dye	λ_Abs_ (nm)	λ_Em_ (nm)	ε (M^−1^ cm^−1^)	Stokes Shift (nm)	Φ_F_	Solvent	Applications
**29**	488	520	---	30	0.89	PBS	H_2_S Sensor
**33**	525	549	122,000	24	0.91	EtOH/TFE	Targeting
**34**	585	609	156,000	24	0.78	EtOH/TFE	Targeting
**35**	635	652	167,000	17	0.56	EtOH/TFE	Targeting
**37**	571	578	---	7	---	ETOH	Al^3+^ Sensor
**38**	515	543	56,000	28	0.86	PBS	Targeting
**39**	496	522	74,000	26	0.89	PBS	Targeting
**40**	515	536	65,000	21	0.58	PBS	Targeting
**44**	490	512	33,450	22	0.91	TFE	Optical **
**45**	491	513	36,340	22	0.93	TFE	Optical **
**46**	504	526	42,130	22	0.88	TFE	Optical **
**48**	664	681	98,000	17	0.74	DCM	NIR *
**50**	695	707	149,000	12	---	EtOH	NIR *

* NIR used for fluorescence imaging compounds*;* ** Optical used for compounds used for fluorescence studies in visible range.

**Table 3:** Optical properties of selected fluorinated phthalocyanine dyes.

Dye	λ_Abs_ (nm)	λ_Em_ (nm)	ε (M^−1^ cm^−1^)	Stokes Shift (nm)	Φ_F_	Solvent	Applications
**51**	712	715	133,000	3	0.77	Toluene	PDT
**54**	678	683	134,900	5	0.13	MeOH	PDT
**55**	694	699	158,500	5	0.11	MeOH	PDT
**56**	676	689	---	13	0.11	DMSO	Optical **
**59**	677	---	---	---	---	DMSO	PDT
**60**	695	---	---	---	---	DMSO	PDT
**63**	675	685	218,000	10	0.52	DMF	Optical **
**64**	675	685	276,000	10	0.43	DMF	Optical **
**66**	695	---	70,795	---	---	---	NH_3_ Sensor
**68**	650	702	---	---	0.09	DMSO	NIR *
**69**	650	676	---	26	0.14	DMSO	NIR *

* NIR used for fluorescence imaging compounds*;* ** Optical used for compounds used for fluorescence studies in NIR range.

**Table 4:** Optical properties of selected fluorinated cyanine dyes.

Dye	λ_Abs_ (nm)	λ_Em_ (nm)	ε (M^−1^ cm^−1^)	Stokes Shift (nm)	Φ_F_	Solvent	Applications
**74**	570	588	38,300	18	0.12	EtOH	NIR *
**77**	656	682	228,000	16	0.29	EtOH	NIR *
**78**	758	790	180,000	32	0.16	EtOH	NIR *
**80**	760	782	---	22	---	PBS/DMSO	NIR *
**81**	805	825	---	20	0.09	DMSO	NIR *
**83**	616	730	38,750	114	0.02	EtOH	NIR *
**84**	619	731	32,950	112	0.01	EtOH	NIR *
**85**	622	732	36,600	110	0.01	EtOH	NIR *
**86**	633	718	51,600	115	0.02	EtOH	NIR *
**87**	651	743	39,000	92	0.05	EtOH	NIR *
**88**	621	732	31,900	111	0.02	EtOH	NIR *
**89**	612	733	43,600	111	0.02	EtOH	NIR *
**93**	---	---	---	---	---	---	NIR *
**94**	---	---	---	---	---	---	NIR *
**104**	804	816	141,000	12	---	MeOH	NIR *
**105**	803	820	256,300	17	---	MeOH	NIR *
**106**	805	817	233,200	12	---	MeOH	NIR *
**109**	692	716	---	24	0.47	PBS	Cys Sensor
**110**	589	660	---	71	---	PBS	Cys Sensor
**112**	560	575	159,471	15	0.18	EtOH	PET
**116**	518	538	126,000	20	0.07	MeOH	PET
**120**	483	636	19,700	153	0.01	DMSO	Optical **
**121**	768	794	---	26	---	MeOH	Targeting
**122**	768	---	---	---	---	MeOH	PET
**128**	641	663	225,800	22	0.32	EtOH	NIR *
**129**	643	658	233,700	15	0.17	EtOH	NIR *
**130**	641	658	210,500	17	0.16	EtOH	NIR *
**131**	638	656	45,200	18	0.59	EtOH	NIR *
**132**	640	656	114,900	16	0.21	EtOH	NIR *
**133**	638	653	49,700	15	0.21	EtOH	NIR *

* NIR used for fluorescence imaging compounds; ** Optical used for compounds used for fluorescence studies in visible range.

## Data Availability

No new data were created or analyzed. Data sharing is not applicable to this article.
